# Antimicrobial and anti-virulent efficacies of melanin, and risk factors associated with *Escherichia coli* and *Salmonella* species from broiler farms and humans: integrating a one health approach

**DOI:** 10.3389/fcimb.2026.1868544

**Published:** 2026-07-20

**Authors:** Amira S. A. Attia, Nashwa El-Gazzar, Hassan Mohmoud Diab, Emad Sakr, Rehab E. Mohamed, Ahmed Al-baqir, Zeinab Saed Ibrahim, Hend Abdalla El-sayed, Ghada Abd Elmoniem Mokhtar, Noura Almadani, Rasha M. M. Abou Elez

**Affiliations:** 1 Department of Veterinary Public Health, Faculty of Veterinary Medicine, Zagazig University, Zagazig, Egypt; 2 Department of Botany and Microbiology, Faculty of Science, Zagazig University, Zagazig, Egypt; 3 Department of Animal and Poultry Health and Environment, Faculty of Veterinary Medicine, Qena University, Qena, Egypt; 4 Department of Animal Hygiene and Zoonoses, Faculty of Veterinary Medicine, University of Sadat City, Sadat City, Menoufia, Egypt; 5 Department of Zoonoses, Faculty of Veterinary Medicine, Zagazig University, Zagazig, Egypt; 6 Department of Avian and Rabbit Medicine, Faculty of Veterinary Medicine, Zagazig University, Zagazig, Egypt; 7 Department of Medical Microbiology and Immunology, Faculty of Medicine, Zagazig University, Zagazig, Egypt; 8 Community and Psychiatric Mental Health Nursing Department, College of Nursing, Princess Nourah Bint Abdulrahman University, Riyadh, Saudi Arabia

**Keywords:** drug resistance, Escherichia coli, gene expression, melanin, Salmonella enteritidis

## Abstract

**Introduction:**

*Esherishia coli* (*E. coli*) and *Salmonella* species (spp.) pose serious public health threats because of their ability to contaminate poultry products and spread through farm environments. This study assessed the prevalence, antimicrobial resistance, and virulence gene profiles of *E. coli* and *Salmonella* spp.

**Methods:**

A cross-sectional study was conducted, involving the random collection of 400 samples from broilers, drinker’s water, feedstuff, broiler litter, and poultry workers in broiler farms. Furthermore, a structural questionnaire was administered to poultry workers to identify potential risk factors and assess the public health implications associated with pathogen transmission. The antimicrobial and anti-virulence activities of melanin against *E. coli* and *Salmonella enteritidis* (*S. enteritidis*) were also assessed.

**Results:**

*E. coli* and *Salmonella* spp. were frequently isolated from broiler farms, with a predominance of. *E. coli* serotype O111:H2 (25.64%) and *S. enteritidis* strains (30.4%) among the total examined samples. Multivariate analysis revealed that poultry workers, who had symptoms worsened after starting farm work and those did not use of personal protective equipment (PPE) were strongly associated with *E. coli* positivity. *E. coli* isolates exhibited full resistance to ampicillin (AMP) and tetracycline (TET), whereas *Salmonella* spp. isolates showed full resistance to AMP. Most of the examined *E. coli* isolates (71.8%) and the *Salmonella* spp. isolates (60.9%) were multidrug resistant (MDR) strains. In addition, 28.2% of *E. coli* isolates and 30.4% of *Salmonella* spp. isolates were -XDR. The *stx*1, *stx*2, and *hyl*A virulence genes were respectively detected in 69.2%, 69.2%, and 74.4% of the examined *E. coli* isolates, whereas the *sop*B and *hil*A virulence genes were identified in all *Salmonella* spp. isolates. One-way repeated-measures analysis of variance (ANOVA) showed that natural melanin treatment significantly reduced the expression of virulence genes in both *E. coli* and *S. enteritidis* isolates.

**Conclusion:**

The predominance of *E. coli* serotypes O111 and *S. enteritidis* highlights the need for improved hygiene practices and zoonotic diseases awareness to limit pathogen transmission in poultry farms. The detection of MDR and XDR strains e emphasizes the consequences of antimicrobial misuse and its implications for public health. Melanin has potential antimicrobial and anti-virulence activities against *E. coli* and *S. enteritidis*.

## 1 Introduction

The poultry industry plays a vital role in supporting global food security, particularly in Egypt. Among livestock production systems, broiler farming represents one of the largest contributors to global meat production because of its short production cycle, high feed conversion efficiency and relatively low cost (WHO, 2023). In Egypt, broiler production reached approximately 1.8 billion broiler birds in 2019, and projections suggest that output could approach 2 billion broilers by 2025. This growth reflects an increase in consumer demand for affordable low-cost animal protein from poultry (Ahmed, 2019).

However, the intensive rearing system commonly applied on poultry farms, which is characterized by high stocking densities and environmental stress, creates ideal conditions for zoonotic disease transmission. Among the most concerning bacterial pathogens are *Escherichia coli* (*E. coli*) and *Salmonella* species (spp.), which pose major threats to public health, food safety and poultry health (EFSA, 2022; WHO, 2023).

Avian colibacillosis and salmonellosis are globally distributed bacterial diseases that cause significant morbidity and mortalityin poultryand heavy economic losses in the poultry industry (Khalefa et al., 2025; Okpalaji et al., 2025. Colibacillosis causes a variety of diseases in poultry, including yolk sac infection, omphalitis, respiratory tract infection, swollen head syndrome, acute colisepticemia, coligranuloma, enteritis, cellulitis, and salpingitis. Similarly, *Salmonella* infection in broilers causes dehydration, gasping, weakness, decreased appetite, lameness, joint swelling, and blindness (Wibisono et al., 2020). The colonization of *E. coli* and *Salmonella* spp. in the intestinal tract of poultry facilitates their persistence and survival in poultry environments, such as soil, litter, and water for a long time and maintains infectivity (EFSA, 2022). Their environmental persistence highlights the urgent need for effective control strategies (Bhattarai et al., 2024; Ramatla et al., 2025).

Human-related factors are considered important contributors to disease transmission within poultry farms. The daily practices of workers, movement of personnel between farms, inadequate training in hygiene measures, protective clothing, and visitor control may facilitate pathogen introduction and spread (Abebe et al., 2020). However, most Egyptian studies have focused mainly on pathogen prevalence and antimicrobial resistance, whereas limited research has evaluated the actual contribution of human-related factors to disease transmission integrated biosecurity strategies or their associations with *E. coli* infections and associated health consequences (Gentile et al., 2023**;** Tilli et al., 2024). Moreover, available information concerning the effectiveness of biosecurity measures in Egyptian poultry production systems and the role of these practices in pathogen dissemination remains insufficiently documented. This gap is partly due to challenges related to workers’ awareness, economic limitations, and farm management conditions (Obe et al., 2023).

In addition to their public health impacts, the antimicrobial resistance (AMR) of *E. coli* and *Salmonella* represents a global challenge. The prolonged and indiscriminate use of antibiotics in poultry production for growth promotion and disease prevention has led to the emergence of resistant bacterial strains and the spread of antibiotic resistance genes (ARGs) (Founou et al., 2016**;** Durrani et al., 2024**;** Oubouyahia et al., 2025). Identifying alternative control strategies for the AMR of *E. coli* and limiting the use of antibiotics constitute a new strategy for preventing and controlling avian colibacillosis (Oubouyahia et al., 2025). Pathogenic AMR of *Salmonella* spp. occurs frequently in poultry environments, predominantly evolving from unregulated infectious wastes containing antimicrobial substances released by poultry farms (Kipre et al., 2025). Farming practices, the physiological conditions of poultry, manure handling and wastewater management all serve as significant vectors for the dissemination of these resistance genes (Chen et al., 2025).

Resistant strains often harbor mobile genetic elements encoding resistance genes against multiple antimicrobial classes, including beta-lactams, tetracyclines, sulfonamides, aminoglycosides, and second- and third-generation cephalosporins (CDC, 2013**;** Poirel et al., 2018). These resistant bacteria can spread vertically through contaminated eggs or meat, consequently entering the human food chain, thus posing a major zoonotic risk (Bhattarai et al., 2024**;** Ramatla et al., 2025). Antimicrobial resistance is directly associated with 1.3 million deaths worldwide each year, highlightingthat a one-health approach that integrates surveillance and control strategies across the animal, human and environmental health sectors is needed (WHO, 2023**;** Cerqueira-Cézar et al., 2025).

The pathogenicity of avian pathogenic *E. coli* (APEC) is associated with its virulence genes, such asthe shiga-like toxins 1 and/or 2 (*stx*1, *stx*2), intimin (*eae*A), and enterohemolysin (*hly*), which enable it to live an extraintestinal life and establishment of the infection (El-Mongy et al., 2018). The *stx*1 and *stx*2 genes inhibit protein synthesis in susceptible eukaryotic cells (Momtaz et al., 2013**;** Exeni et al., 2018). The *eae*A gene enables the attachment of bacteria to intestinal epithelial cells and inhibits protein synthesis in sensitive eukaryotic cells, leading to cellular damage (Friedrich et al., 2002**;** Luna-Gierke et al., 2014**;** Wei et al., 2020). Furthermore, the *hly*A gene is considered an effective virulence factor in the pathogenicity of *E. coli* (Smith and Fratamico, 2017).

The chromosomes and plasmids of *Salmonella* spp. contain different virulence genes, such as *Salmonella* outer proteins (*sop*s), hyperinvasive locus A (*hil*A), enterotoxin (*stn*), and *Salmonella* plasmid virulence (*spv*s) genes. They play roles in the invasion, survival, and replication of bacteria inside the host, enterotoxin production, and diarrhea in the host (Fluit, 2005**;** Huehn et al., 2010**;** Elemfareji and Thong, 2013**;** Abou Elez et al., 2021). Therefore, there is an urgent need to develop effective natural antimicrobial agents, such as nanoparticles, probiotics, melanin, and essential oils as sustainable alternatives to conventional antibiotics for the control of pathogenic and antimicrobial-resistant bacterial infections (Kumar et al., 2024**;** Myktybayeva et al., 2024; Ike et al., 2025). Therefore, there is an urgent need to develop effective natural antimicrobial agents as sustainable alternatives to conventional antibiotics for the control of pathogenic and antimicrobial-resistant bacterial infections.

Melanin is a natural pigment produced by many microorganisms, plants and animals. It has multiple biological functions, including antioxidant, antimicrobial and inhibitory effects on biofilm formation by Gram-negative bacteria, including *E. coli* and *Salmonella* spp. Recently, emerging evidence has shown that both natural and synthetic forms of melanin can reduce bacterial virulence and enhance the host defense mechanism by disrupting bacterial membrane stability and modulating resistance gene expression (Guo et al., 2023**;** Kumar et al., 2024). Therefore, the application of melanin as a natural, biologically safe, sustainable supplement to antibiotics was introduced in poultry production. Melanin-based approaches may provide an innovative complementary method to control AMR while maintaining poultry health and food safety (Kumar et al., 2024**;** Mahfouz et al., 2025).

This study was conducted to identify the risk factors influencing the prevalence of pathogenic *E. coli* and *Salmonella* spp. isolated from different samples collected from poultry farms in Sharkia Governorate, Egypt. Antimicrobial susceptibility testing and MDR profiles of the recovered *E. coli* and *Salmonella* isolates were also assessed. Additionally, quantitative reverse transcription PCR (RT–qPCR) analysis was performed to evaluate the expression of virulence genes in melanin-treated *E. coli* and *S. enteritidis*.

## 2 Materials and methods

### 2.1 Study design and sampling

**Figure 1:**
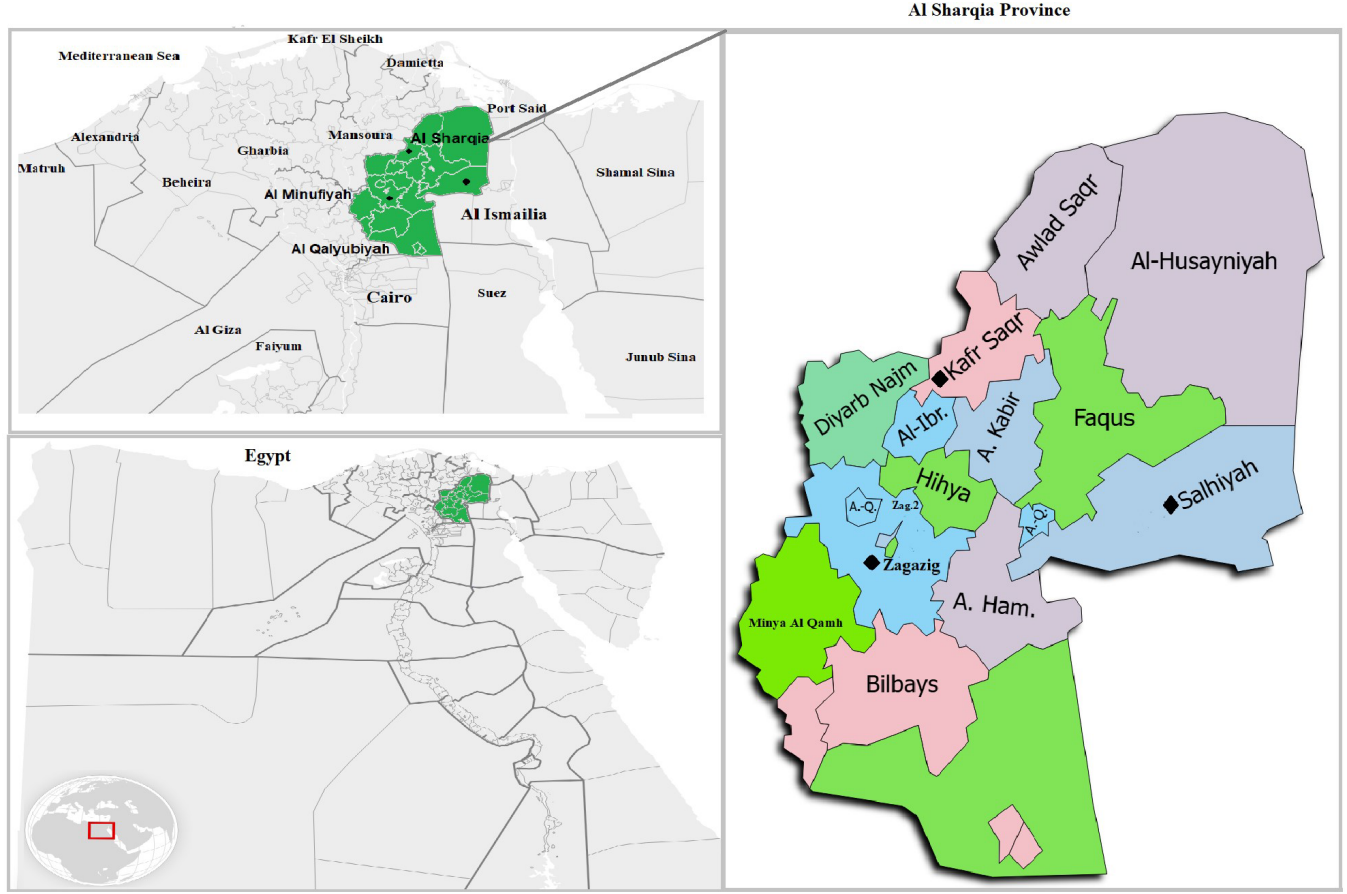
Study area map of commercial broiler poultry farms located across Sharkia Governorate (El-Gar, El- Salhiyah, Kafer Saker), Egypt Geographical Information System (GIS) data were collected from paint maps (http://www.paintmaps.com) and (http://app.datawrapper.de/ignxbvhdnb/select/map).

A cross-sectional investigation was carried out from April to August 2025 in Sharkia Governorate, Egypt. A total of 400 samples were obtained randomly from three commercial broiler poultry farms (El-Gar, El-Salhiyah, and Kafer Saker) located in Sharkia Governorate in the eastern Nile Delta of Egypt, as presented in **Figure 1**. The farm sizes ranged from 5, 000–10, 000 Cobb broilers, with a stocking density of 8–10 birds/m2, aged 1–45 days, and reared under semi-closed housing systems. All farms were climate–controlled, naturally ventilated through hopper-style windows (22–30 per side), and equipped with electrical fans. The main water supply of the farms was underground water. The selection of broiler farms was based on their owners’ willingness to permit random sampling methods, following the guidelines proposed by Bellhouse (2005). Each farm was visited three times for sample collection. A group of 180 apparently healthy broilers showing no clinical signs of infection were randomly selected. From each farm, 30 drinker water samples, 12 poultry feed samples, 18 litter samples, and 60 cloacal swabs were collected. Additionally, 40 hand swabs were obtained from poultry workers. Cloacal and hand swabs were collected aseptically via sterile swabs immersed in 9 milliliters (mL) of pre-enrichment buffered peptone water ((BPW; Oxoid Ltd., Basingstoke, Hampshire, UK), following the methods of Abunna et al. (2016) and Abdi et al. (2017). Litter samples (the top dry and wet layers) and 100 grams (g) of well-mixed homogenized feedstuff were collected aseptically via sterile plastic spoons and zip-lock bags. For drinker water sampling, 100 mL was collected from each poultry farm in sterile screw-capped colorless glass vials (Zaki et al., 2023; Attia et al., 2024). All collected samples were properly labeled with the farm name, sample type, and collection date, then placed in an insulated ice box (4 ± 1 °C), and transported aseptically immediately to the laboratory of the Veterinary Public Health Department, Faculty of Veterinary Medicine, Zagazig University, for bacteriological examination. Processing was performed within six hours in accordance with ISO 7218:2007 standards (ISO, 2013).

### 2.2 Isolation and identification of *E. coli* and *Salmonella* spp.

Approximately 25 mL/or g of each water, feedstuff or litter samples were homogenized in 225 mL of sterile -BPW (Arthur et al., 2004), while the cloacal and human hand swabs were immersed in 9 mL of BPW (Cruichshank et al., 1975). The pre-enriched broths were incubated aerobically overnight at 37 °C for 24 hours (h). To isolate *E. coli, a* loopful of each pre-enriched broth was transferred to 10 mL of MacConkey broth and incubated for 24 h at 37 °C. A loop from each positive broth was subsequently streaked over eosin methylene blue (EMB) agar (Oxoid™ CM0069B), and incubated for 24 h at 37 °C. For *Salmonella* spp. isolation, a loopful of the pre-enriched broth was inoculated into 10 mL of Rappaport Vassiliadis soya (RV) broth (Difco, USA), and incubated for 24 h at 42 °C. A loop of positive RV broth was streaked on xylose lysine deoxycholate (XLD) agar (Oxoid™ CM0469) and incubated at 37 °C for 24 h (Humphrey et al., 1989**).** Typically, *E. coli* colonies appeared as small metallic green shiny colonies on EMB agar, whereas *Salmonella* colonies presented translucent red colonies with black centers. The presumptive colonies were picked up for purification on tryptone soya agar (Oxoid, UK) and subjected to biotyping and serotyping. The suspected isolates were examined morphologically via Gram staining and confirmed biochemically via indole, sugar fermentation, methyl red, triple sugar iron, Voges–Proskauer, and catalase tests (Sobur et al., 2019**;** Zaman et al., 2020**;** Ievy et al., 2020**).** Serological identification of *E. coli* and *Salmonella* spp. isolates was performed via rapid diagnostic antisera kits (DENKA SEIKEN Co., Japan) according to the White–Kauffmann technique (Kauffmann, 1974**).** Slide agglutination test with polyvalent and then monovalent antisera based on somatic (O) and flagellar (H) antigens were generated as described by Grimont and Weill (2007). Suspected *E. coli* and *Salmonella* isolates were kept frozen at -80 °C in brain heart infusion (BHI) broth (Oxoid, UK) and were supplemented with 15% glycerin (Synth^®^, Brazil) for further examination.

### 2.3 Risk factors associated with the prevalence of *E. coli* and *Salmonella* spp.

A structured, face-to-face interview was conducted using a pre-designed questionnaire consisting of 21 close ended-questions. A total of 40 questionnaires were administered for data collection. The questionnaire was developed by the researchers with reference to previously published studies by Sharma et al. (2019) and Attia et al. (2024). Prior to data collection, the questionnaire was pre-tested among poultry workers to ensure clarity and appropriateness for addressing the study objectives. The questionnaire comprised four main sections. The first section focused on socio-demographic characteristics and occupational background of the farm owners and poultry handlers (seven questions). The second section evaluated biosecurity knowledge and training (five questions). The third section explored aspects of human health (five questions). The final section examined hygiene practices and safety measures (four questions). All participants were informed about the study’s purpose, and verbal consent was obtained prior to participation. Confidentiality was maintained throughout the process, and individuals who refused to participate were excluded from the study.

### 2.4 Antimicrobial susceptibility test of *E. coli* and *Salmonella* spp. isolates

All serologically identified *E. coli* isolates (n=39) and *Salmonella* spp. isolates (n=23) were tested for susceptibility to 21 antibiotics belonging to 15 different classes via the Kirby–Bauer disk diffusion method following the Clinical and Laboratory Standards Institute (CLSI) guidelines (2018). Twenty-one antibiotic disks (Thermo Fisher Scientific, Oxoid Ltd., UK) were used: gentamicin (CN, 10 µg), tobramycin (TOB, 10 µg), amikacin (AK, 30 µg), piperacillin-tazobactam (PPT, 30 µg), imipenem (IPM, 10 µg), cefazolin (CFZ, 30 µg), cefotaxime (CTX, 30 µg), ceftazidime (CAZ, 30 µg), cefepime (FEP, 30 µg), cefoxitin (CFT, 30 µg), ciprofloxacin (CIP, 5 µg), nalidixic acid (NAL, 30 µg), trimethoprim-sulphamethoxazole (SXT, 1.25/23.75 µg), aztreonam (ATM, 30 µg), ampicillin (AMP, 10 µg), ampicillin–clavulanic acid (SAM, 30 µg), chloramphenicol (CHL, 30 µg), colistin (CT, 25 µg), tetracycline (TET, 30 µg), nitrofurantoin (NIT, 300 µg) and azithromycin (AZM, 30 µg). Briefly, the bacterial suspension was prepared in sterile saline and adjusted to the turbidity of the 0.5 McFarland standard. The suspension (150 µL) was then inoculated on Muller–Hinton agar plates (MHA) (Thermo Fisher Scientific, Oxoid Ltd., UK) via sterile swabs. Antibiotic discs were placed on the surface of the plates, which were subsequently incubated at 37 °C for 18–24 h. *E. coli* ATCC 25922 used as a reference control strain. The inhibition zone diameter was measured, and interpreted according to the CLSI criteria, and was reported as resistant (R), intermediate (I) and sensitive (S). Multiple antibiotic resistance (MAR) was determined via the following formula: a/b (where “a” is the number of antimicrobial agents to which an isolate was resistant and “b” is the total number of antimicrobial agents tested)) following the protocol proposed by Krumperman (1983). The interpretation of antibiotic susceptibility to evaluate MDR, extensive drug resistant (XDR), and pandrug resistant (PDR) isolates was grouped according to the CLSI criteria and Magiorakos et al. (2012). Therefore, the isolates that exhibited non-susceptibility to at least one agent in ≥ 3 antimicrobial classes were considered MDR, the isolates that exhibited non-susceptibility to at least one agent in ≥ 6 antimicrobial classes were considered XDR, and the isolates that exhibited non-susceptibility to all agents in all antimicrobial classes were considered PDR.

### 2.5 Molecular identification and virutyping of *E. coli* and *Salmonella* spp. isolates

Genomic DNA was extracted from 39 *E. coli* and 23 *Salmonella* spp. isolates via the QIAamp DNA Mini Kit (QIAGEN GmbH, Germany), following the manufacturer’s guidelines by uniplex PCR targeting the 16S *rRNA* (Seidavi et al., 2010**),** and *inv*A genes, respectively (Oliveira et al., 2003). PCR-confirmed *E. coli* isolates were screened for the detection of *stx*1 (Dipineto et al., 2006), *stx*2 (Dipineto et al., 2006), *eae*A (Mazaheri et al., 2014), and *hyl*A (Fratamico et al., 1995). The PCR-confirmed *Salmonella* spp. were screened for the detection of the *sop*B (Huehn et al., 2010), *stn* (Murugkar et al., 2003), *spv*C (Huehn et al., 2010), and *hil*A (Yang et al., 2014) virulence genes. **Supplementary Table 1** displays the sequences of primers and cycling condition used foreach gene in this study. The PCR mixture (25 microliters) (µL) for each tested gene consisted of 2x premix Emerald Amp GT PCR master mix (12.5 µL) (Takara Bio Inc., Shiga, Japan), PCR-grade water (4.5 µL), forward and reverse primers (µL each), and template DNA (1 µL). DNA amplification was performed in a T3 thermal cycler (Biometra). Positive *S. enteritidis* ATCC 13076 and *E. coli* ATCC 25922 control strains donated by the Biotechnology Unit, Reference Laboratory for Veterinary Quality Control on Poultry Production, Animal Health Research Institute, Dokki, Giza, Egypt, were run alongside the tested isolates. The amplified products, positive controls (*S. enteritidis* ATCC 13076 and *E. coli* ATCC 25922), and negative controls for each target gene, along with a DNA ladder (Fermentas), were loaded onto an ethidium bromide (0.5 micrograms/mL) (µg/mL)-stained agarose gel (1.5%) and run for 30 minutes (min). The resulting gel was photographed with a gel documentation system (Alpha Innotech), and the data were analyzed via computer software.

### 2.6 Antimicrobial activity of natural melanin

The naturally purified melanin extracted previously from *Aspergillus flavus* (*A. flavus*), accession number MZ314535, was used (El-Gazzar et al., 2025) and tested against two selected XDR *E. coli* and XDR *S. enteritidis* isolates recovered from broilers, which were positive for all the virulence genes tested in this study. The disk diffusion agar method was used to test the antimicrobial activity of natural melanin (Bauer, 1966). A melanin suspension at a concentration of 1 mg/mL was prepared by suspending 10 mg of purified melanin in 10 mL of 1% pure dimethyl sulfoxide (DMSO; Sigma-Aldrich, USA) and used as a stock solution for further experiment. A total of 10 µg of the prepared melanin was placed on standard disks with a diameter of 6 mm (Padtanteb, Qods, Iran). The *E. coli* and *S. enteritidis* isolates were spread on MHA plates. The disks were placed on the agar plates, and NIT disks (300 µg) and sterile blank disks were used as controls, then the inhibition zone was measured after 24 h incubation at 37°C. The diameters of the inhibition zones were measured via a metric ruler. The experiment was performed in triplicate.The minimum inhibitory concentration (MIC) and minimum bactericidal concentration (MBC) of natural melanin were determined via the tube dilution method as previously described by Krishnan et al. (2015). The bacterial inocula were adjusted to match a 0.5 McFarland turbidity standard. Purified melanin was prepared by making two-fold serial dilutions in Mueller–Hinton broth (MHB) (Merck, Darmstadt, Germany), with concentrations ranging from 0.1 mg/mL to 3.2 mg/mL. Aproximatelyt 100 µL of melanin (dissolved in 1% DMSO) at different concentrations (0.1, 0.15, 0.2, 0.3, 0.4, 0.6, 0.8, 1.2, 1.6, 2.4, and 3.2 mg/mL) was added to 100 µL from the tested organism and 5 mL of MHB, and then incubated at 37°C with shaking for 24 h. The broth media without melanin was used as the negative control. The positive control consisted of 100 μL of NIT solution and 100 μL of bacterial suspension. The MIC is the lowest concentration of melanin that shows no visible turbidity after further 24 h. The MBC was determined by coating the bacterial inoculum with different melanin suspensions on the MHA plate and then incubating it at 37 °C for 24 h. The MBC value was defined as the lowest concentration, with no visible growth on the MHA plate.

### 2.7 Virulence gene expression of melanin-treated *E. coli* and *S. enteritidis*

#### 2.7.1 Bacterial counting

Two XDR isolates (*E. coli* and *S. enteritidis*) were selected for this experiment, and these isolates were positive for all the virulence genes tested in this study. The bacterial inoculum was adjusted to 1.5×10^8^ CFU/mL and inoculated at a final concentration of 0.3 mg/mL for *E. coli* and 0.2 mg/mL for *S. enteritidis*, followed by constant stirring to obtain a uniform colloidal melanin suspension via the surface plating method on MHA plates (Thatcher and Clark, 1968) for bacterial counting. Broth media without melanin (100 μL) and bacteria-free suspension (100 μL) were used as the control positive and negative, respectively. Each inoculated suspension was incubated at 37 °C for 24 h, 48 h, 72 h, 96 h, and 120 h. The inhibition of cell growth was determined by counting the number of CFUs on the plates via the surface plating method. Bacteria and melanin mixtures were prepared at different cultivation times and were verified via amplification of the 16S *rRNA* gene via PCR. Bacterial counts were measured in triplicate and are expressed as the mean values and standard deviations.

#### 2.7.2 RT–qPCR analysis of gene expression

To protect RNA from degradation, one volume of the harvested bacterial culture was added to one volume of RNA Protect Bacteria Reagent (Qiagen, Germany, GmbH) at each incubation time and processed following the manufacturer’s guidelines. The RNA isolation and cDNA synthesis were implemented via the QIAamp RNeasy Mini Kit (Qiagen, Germany) following the manufacturer’s instructions. The melanin *E. coli* isolate was screened for the expression of the *stx*1, *stx*2, *eae*A, and *hyl*A virulence genes, and the melanin-treated *S. enteritidis* isolate was screened for the expression of the *sop*B, *stn*, *spv*C, and *hil*A virulence genes (all of which are exhibited in one isolate used) via SYBR Green I-based real-time PCR with specific primers for each gene as previously described, and the 16S *rRNA* gene was used as a housekeeping gene (Yang et al., 2014). The primers were utilized in a 25-µL reaction containing 12.5 µL of 2× QuantiTect SYBR Green PCR master mix (Qiagen), 0.25 µL of RevertAid reverse transcriptase (200 U/µL) (Thermo Fisher), 0.5 µL of each primer (20 pmoL concentration), 8.25 µL of water, and 3 µL of RNA template. The reaction was performed in a Stratagene MX3005P real-time PCR machine. The reaction conditions for tested genes were presented in **Supplementary Table 1**, and a house keeping 16S *rRNA* gene included reverse transcription at 50 °C for 30 min, primary denaturation at 94 °C for 15 min, and 40 cycles of denaturation at 94 °C for 15 seconds (s), annealing at 62 °C for 30 s, and extension at 72 °C for 30 s. The melting curve was performed after all cycles of amplification and fluorescence reading was performed at every degree between 55 °C and 95 °C to ensure that only one PCR product was amplified. Amplification curves and cycle threshold (Ct) values were determined by Stratagene MX3005P software. The variations in RNA gene expression of the different samples were measured. The comparative Ct method was compared between each sample’s Ct and the positive control group. The ΔΔCt method was performed, according to Yuan et al. (2006).

### 2.8 Statistical analysis

Statistical analyses were performed using SPSS version 25 (IBM, Armonk, NY, USA). Categorical variables were summarized as frequencies and percentages, while continuous variables were expressed as means ± standard deviations. Chi-square or Fisher’s exact tests (cell counts were <5) were applied to explore how *E. coli* and *Salmonella* spp. were distributed across different risk factors. Pearson correlation analysis was performed to assess relationships in pathogen isolation rates across different sample sources, and correlation coefficients (r) were weak (0.1–0.3), moderate (0.3–0.5), or strong (0.5–1.0). Univariate logistic regression analysis was conducted to estimate odds ratios (ORs) with 95% confidence intervals (CIs) for each potential risk factor. Variables with P < 0.05 in univariate analysis were entered into a multivariable logistic regression model to identify independent predictors of bacterial isolation, and adjusted odds ratios with 95% CIs were reported. Model assumptions, including independence of observations and absence of multicollinearity, were assessed before analysis. Wide confidence intervals observed in some regression estimates were attributed to the relatively small number of positive cases across categorical groups (Pallant, 2011). For the gene expression study, relative m*RNA* expression levels of *E. coli* (*stx*1, *stx*2, *eae*A, and *hyl*A) and *S. enteritidis* (*sop*B, *stn*, *hil*A, and *spv*C) virulence genes following treatment with natural melanin at different incubation times (24, 48, 72, 96, and 120 h) were analyzed using one-way repeated-measures analysis of variance (ANOVA). When significant overall differences were detected, Bonferroni-adjusted *post hoc* multiple comparisons were performed to identify differences between incubation times. Statistical significance was set at P < 0.05 for all analyses.

## 3 Results

### 3.1 Prevalence of *E. coli* and *Salmonella* spp.

A total of 400 samples were collected from various sources, including broiler cloacal swabs, drinking water, litter, feedstuff, and poultry workers’ hand swabs, to assess the presence of *E. coli* and *Salmonella* spp. (**Table 1**). Among these strains*, E. coli* was identified in 207, representing a prevalence rate of 51.75%. The highest prevalence was recorded in cloacal swabs (61.67%), followed by water (54.44%), litter (44.44%), poultry workers (37.5%), and feedstuff (22%). The chi-square test revealed a statistically significant association only between cloacal swabs and litter, as presented in **Table 1**.

**Table 1:** Prevalence of *E. coli and Salmonella* spp. isolated from different sources collected from poultry farms.

Source of sample	No. of samples	No. of Positive *E. coli* (%)1	No. of positive *Salmonella* spp. (%)1	No. of positive *Salmonella* serovars (%)2						
				*S. enteritidis*	*S. Kentukey*	*S. typhimurium*	*S. Wingrove*	*S. Bargny*	*S. anatum*	*S. Virchow*
Cloacal swabs	**180**	111 (61.67)^a^	18 (10)^a^	5 (31.6)	2 (10.5)	2 (15.8)	3 (15.8)	1 (5.3)	3 (21.1)	2 (10.5)
Drinkers’ water	**90**	49 (54.44)^a,b^	1 (1.1)^a^	1 (50)	0 (0.0)	0(0.0)	0 (0.0)	0 (0.0)	0 (0.0)	0 (0.0)
Litter	**54**	24(44.44)^b^	2 (3.7)^a^	0 (0.0)	0 (0.0)	1(50)	0 (0.0)	0 (0.0)	1 (50)	0 (0.0)
Workers’ hand swabs	**40**	15 (37.5)^a,b^	2 (5)^b^	1 (50)	0 (0.0)	1 (50)	0 (0.0)	0 (0.0)	0 (0.0)	0 (0.0)
Feedstuff	**36**	8 (22)^a,b^	0 (0.0)^a^	0 (0.0)	0 (0.0)	0 (0.0)	0 (0.0)	0 (0.0)	0 (0.0)	0 (0.0)
Total	**400**	**207 (51.7 5)**	**23 (5.75)**	**7 (30.4)**	**2 (8.7)**	**4 (17.4)**	**3 (13.04)**	**1 (4.3)**	**4 (17.4)**	**2 (8.7)**

^1^ calculated according to the number of examined samples.

^2^ calculated according to the number of positive samples.

^a,b^ different letters indicate statistically significant level of chi-square value at P < 0.05.

*Salmonella* spp. were detected in 23 samples (5.75%), with the highest prevalence in cloacal swabs (10%), followed by poultry workers (5%), litter (3.7%) and drinking water (1.1%). No *Salmonella* spp. were detected in the feed samples. A statistically significant association was observed between the levels of *Salmonella* in the litter and workers’ hand swabs as shown in **Table 1**.

**Figure 2:**
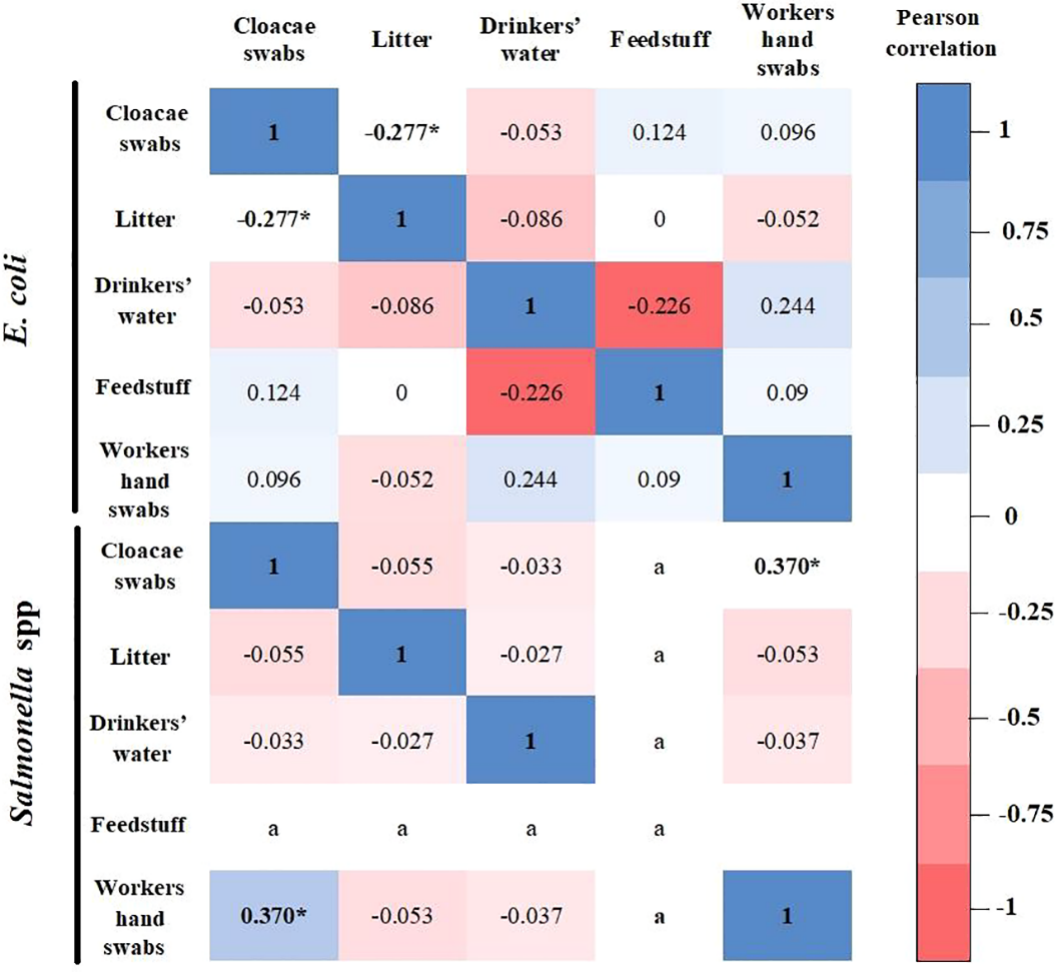
A Pearson correlation coefficient heat map for *E. coli* and *Salmonella* spp. Inter-relationship between different sources in poultry farms. The scale on the right-hand side indicates the correlation coefficient values, with 1.0 representing a perfect positive correlation, −1.0 a perfect negative correlation, and 0 no correlation.

In **Table 2** and **Figure 2**, there was a statistically significant but weak negative correlation between *E. coli* in cloacal swabs and that in litter samples (r = –0.277, *P* = 0.042). In contrast, a weak positive correlation was observed between cloacal swabs and poultry workers’ hand swabs for *Salmonella* spp. (r = 0.370, *P* = 0.019).

**Table 2:** A Pearson correlation coefficient for *E. coli* and *Salmonella* spp. inter-relationship from different sources in examined poultry farms.

Pathogens	Sources	Cloacal swabs	Litter	Drinkers’ water	Feedstuff	Workers’ hand swabs
*E. coli*	Cloacal swabs	1				
	Litter	-0.277*	1			
	Drinkers’ water	-0.053	-0.086	1		
	Feedstuff	0.124	0.000	-0.226	1	
	Workers’ hand swabs	0.096	-0.052	0.244	0.090	1
*Salmonella* spp.	Cloacae swabs	1			a	
	Litter	-0.055	1		a	
	Drinkers’ water	-0.033	-0.027	1	a	
	Feedstuff	a	a	a	a	a
	Workers’ hand swabs	0.370*	-0.053	-0.037	a	1

*: Statistically significant at P-value< 0.05.

a: Indicate correlation cannot be computed because at least one of the variables is constant.

A weak correlation ranges from 0.1–0.3, moderate from 0.3–0.5, and strong from 0.5–1.0.

### 3.2 Serotyping of *E. coli* and *Salmonella* spp. isolates

Among the 39 biochemically confirmed *E. coli* isolates, nineteen distinct serotypes were identified from broilers, drinking water, litter, feedstuff and poultry workers (**Table 1**). The most frequent serotypes detected were *E. coli* O111:H2 (25.64%), and *E. coli* O127:H6 (10.25%), both of which were recovered from broilers and poultry workers.

Similarly, the twenty-three *Salmonella* spp. isolates obtained were serologically classified into seven serotypes, including S*. enteritidis*, *S. kentukey*, *S. typhimurium*, *S. wingrove*, *S. bargny*, *S. anatum*, and *S. virchow* (**Table 1**). *S. enteritidis, S. typhimurium*, and *S. anatum* were the most frequently detected bacteria in broilers, with prevalence rates of 30.4%, 17.4% and 17.4%, respectively. Notably, *S. enteritidis* was the predominant serotype recovered from the broilers, drinking water and poultry workers’ hand swabs, as detailed in **Table 1**.

### 3.3 Factors associated with the prevalence of *E. coli* and *Salmonella* spp. in broiler farms

**Table 3** outlines the isolated *E. coli* and *Salmonella* spp. associated with univariate and multivariate logistic regression analysis of risk factors in the surveyed poultry farms. According to the “socio-demography” data, only the education level of the participants on the farmwas significantly associated with *E. coli* positivity. Respondents with primary education and those who were illiterate only had higher odds of *E. coli* positivity, being 8.75 and 10.50 times more likely to be positive compared with graduates statistically significant (*P* = 0.018 for primary education; *P* = 0.032 for illiterate). On the other hand, no significant association was observed between education level and *Salmonella* spp. prevalence.

**Table 3:** Univariate logistic regression analysis of risk factors associated with isolated pathogens in investigated poultry farms.

Risk factors	Total N (%)	Positive for bacterial pathogens (%)							
		*E. coli*							*Salmonella* spp.
		N (%)	OR	95% CI	P-value	N (%)	OR	95% CI	P-value
I- Demographics and work history									
1. Age									
• ≤35 years	13 (32.5)	4 (30.77)	0.111	0.01, 1.34	0.083	1 (7.69)	1.3x10^8^	0.00	0.999
• 36–50 years	22 (55.0)	7 (31.81)	0.117	0.01, 1.25	0.075	1 (4.5)	7.6 x10^7^	0.00	0.999
• ≥51 years	5 (12.5)	4 (80)	r			0 (0.0)	r		
2. Gender									
• Male	30 (75)	11 (36.66)	0.868	0.20, 3.77	0.850	1 (3.33)	0.310	0.02, 5.48	0.424
• Female	10 (25)	4 (40)	r			1 (10)	r		
3. How long do have you worked in poultry (experience in poultry) production?									
• ≤5 years	14 (35)	5 (35.71)	0.556	0.03, 10.93	0.699	1 (7.14)	1.2 x10^8^	0.00	0.999
• ≤10 years	16 (40)	6 (37.5)	0.600	0.03, 11.47	0.734	1 (6.25)	1.1 x10^8^	0.00	0.999
• ≥11 years	8 (20)	3 (37.5)	0.600	0.03, 13.58	0.748	0 (0.0)	1.000	0.00	1.000
• No experience	2 (5)	1 (50)	r			0 (0.0)	r		
4. Position on the farm									
• Farm owner/manager	4 (10)	2 (50)	2.000	0.18, 22.06	0.571	0 (0.0)	1.000	0.00	1.000
• Farm worker	16 (40)	8 (50)	2.000	0.37, 10.92	0.423	2 (12.5)	2.3 x10^8^	0.00	0.999
• Veterinarian & Physician	6 (15)	0 (0.0)	0.000	0.000	0.999	0 (0.0)	1.000	0.00	1.000
• Catcher	5 (12.5)	2 (40)	1.333	0.14, 12.82	0.803	0 (0.0)	1.000	0.00	1.000
• Employ & other	9 (22.5)	3 (33.3)	r			0 (0.0)	r		
5. Full-time or part-time employee?									
• Full-time	23 (57.5)	10 (43.47)	0.769	0.09, 6.45	0.809	2 (8.69)	1.5 x10^8^	0.00	0.999
• Part-time	13 (32.5)	3 (23.07)	0.300	0.03, 3.13	0.315	0 (0.0)	1.000	0.00	1.000
• Other	4 (10)	2 (50)	r			0 (0.0)	r		
6.Education level									
• Illiterate	9 (22.5)	5 (55.55)	8.750	1.21, 63.43	0.032*	1 (11.11)	2.0 x10^8^	0.00	0.998
• Primary	10 (25)	6 (60)	10.500	1.50, 73.67	0.018*	1 (10)	1.8 x10^8^	0.00	0.998
• Secondary	5 (12.5)	2 (40)	4.667	0.46, 47.63	0.194	0 (0.0)	1.000	0.00	1.000
• Graduate	16 (40)	2 (12.5)	r			0 (0.0)	r		
7. Which tasks do you regularly perform? (Select all that apply)									
• Feeding & watering birds	9 (22.5)	6 (66.66)	0.429	0.04, 4.64	0.486	1 (11.11)	1.000	0.00	1.000
• Vaccination & medical care	9 (22.5)	1 (25)	1.200	0.13, 11.05	0.872	0 (0.0)	1.000	0.00	1.000
• Cleaning & disinfecting	7 (17.5)	2 (28.57)	1.125	0.11, 11.60	0.921	1 (14.28)	2.7 x10^8^	0.00	0.999
• Administrative work & other	10 (25.0)	3 (37.5)	1.000	0.11, 8.95	1.000	0 (0.0)	1.8 x10^8^	0.00	0.999
• Catching birds	5 (12.5)	2 (40)	r				r		
II- Biosecurity knowledge and training									
1. Have you received formal biosecurity training?									
• Yes	9 (22.5)	2 (22.22)	0.396	0.07, 2.22	0.292	0 (0.0)	0.00	0.00	0.999
• No	31 (77.5)	13 (41.9)	r			2 (6.25)	r		
2. If yes, how often is it refreshed or repeated?									
• Annually	4 (44.44)	0 (0.0)	0.000	0.00	0.999	0 (0.0)	-	-	-
• Once	5 (55.55)	2 (40)	r			0 (0.0)	r		
3. Do you know the mode of diseases transmission on a poultry farm?									
• Yes	24 (60)	8 (33.33)	0.643	0.17, 2.36	0.506	0 (0.0)	0.00	0.00	0.998
• No	16 (40)	7 (43.75)	r			2 (12.5)	r		
4. Are you aware on zoonotic diseases?									
• Yes	29 (72.5)	11 (37.93)	1.069	0.25, 4.51	0.927	0 (0.0)	0.00	0.00	0.998
• No	11 (27.5)	4 (36.36)	r			2 (18.18)	r		
5. If yes, what diseases were covered? (Select all that apply)									
• Avian Influenza	1 (3.45)	0 (0.0)	0.000	0.00	1.000	0 (0.0)	-	-	-
• Salmonella	5 (17.24)	0 (0.0)	0.000	0.00	0.999	0 (0.0)	-	-	-
• Campylobacter	1 (3.45)	0 (0.0)	0.000	0.00	1.000	0 (0.0)	-	-	-
• E.coli	13 (44.83)	7 (53.85)	1.458	0.26, 8.05	0.665	0 (0.0)	-	-	-
• Both *Salmonella* & *E.coli*	9 (31.03)	4 (44.44)	r			0 (0.0)	-	-	-
III- Human Health reports									
1. In the last 48 h., have you had contact with any other birds or other livestock anywhere?									
• Yes	14 (35)	6 (42.88)	0.706	0.19, 2.67	0.608	1 (7.14)	1.923	0.11, 33.30	0.653
• No	26 (65)	9 (34.6)	r			1 (3.85)	r		
2. Do you have any pre-existing health conditions?									
• Yes	23 (57.5)	11 (47.82)	2.979	0.74, 11.93	0.123	2 (8.69)	1.5 x10^8^	0.00	0.998
• No	17 (42.5)	4 (23.53)	r			0 (0.0)	r		
3. If yes, currently suffer from any of the following? (Select all that apply)									
• Respiratory symptoms	4 (17.39)	1 (25)	0.167	0.01, 2.37	0.186	0 (0.0)	0.000	0.00	0.999
• Skin symptoms	4 (17.39)	3 (75)	1.500	0.11, 21.31	0.765	0 (0.0)	0.000	0.00	0.999
• Gastrointestinal symptoms	6 (26.08)	1 (16.66)	0.100	0.01, 1.29	0.077	1 (25)	1.600	0.08, 31.77	0.758
• Mixed infection	9 (39.13)	6 (66.66)	r			1 (14.28)	r		
4. Did any symptoms start or worsen when you began working on the farm?									
• Yes	11 (27.5)	9 (81.8)	17.250	2.92, 101.90	0.002*	1 (8.33)	2.800	0.16, 49.10	0.481
• No	29 (72.5)	6 (20.7)	r			1 (3.57)	r		
5. Did use antibiotic in the last three months?									
• Yes	23 (57.5)	11 (47.83)	2.979	0.74, 11.93	0.123	2 (8.69)	1.5 x10^8^	0.00	0.998
• No	17 (42.5)	4 (23.53)	r			0 (0.0)	r		
IV- Hygiene knowledge and practices									
1. How often do you wash or sanitize your hands while at work?									
• Only when they look dirty	7 (20)	7 (100.0)	2.7 × 10^18^	0.000	0.998	1 (20)	2.7 x10^8^	0.00	0.999
• Only after touching birds…	10 (25)	0 (0.0)	1.000	0.000	1.000	1 (12.5)	1.8 x10^8^	0.00	0.999
• At the end of workday	10 (25)	8 (80)	6.5× 10^9^	0.000	0.998	0 (0.0)	1.000	0.00	1.000
• In all above cases	13 (32.5)	0 (0.0)	r				r		
2. Do you eat, drink, or smoke in poultry areas?									
• Yes	10 (25)	9 (90)	36.00	3.79, 342.02	<0.001*	0 (0.0)	3.222	0.18, 56.88	0.424
• No	30 (75)	6 (20)	r			2 (6.66)	r		
3. Do you always use personal protective equipment (PPE) when work with birds?									
• Yes	29 (72.5)	8 (27.59)	0.218	0.050, 0.950	0.043*	1 (3.44)	0.357	0.02, 6.26	0.481
• No	11 (27.5)	7 (63.64)	r			1 (9.09)	r		
4. If yes, do you clean/changed PPE (especially) boot or gloves between houses?									
• Yes	13 (44.8)	1 (7.7)	0.107	0.01, 1.03	0.053	0 (0.0)	0.000	0.000	0.999
• No	16 (55.2)	7 (43.8)	r			2 (25)	r		

*Statistically significant at P-value< 0.05. r, reference; no measures of association are computed because variable is a constant.

The questionnaire findings indicated knowledge gaps in biosecurity (22.5% awareness) and disease transmission routes (60% awareness) with no statistically significant association with pathogens positivity. A higher detection rate of *Salmonella* spp. was observed among participants unaware of zoonotic diseases (18.18%) with no significant association. Concerning pre-existing health conditions, 57.5% of participants were affected, showing higher *E. coli* (47.82%) and *Salmonella* spp. (8.69%) positivity. Among those with pre-existing conditions, symptoms included gastro-intestinal symptoms (26.08%), respiratory/skin (17.39%) and mixed symptoms (39.13%). The highest prevalence of *E. coli* was recorded in mixed symptom cases (66.66%), while *Salmonella* spp. were most frequent in gastrointestinal cases (25%) with no statistically significant associations.

Regarding, symptoms began or worsened after starting farm work, 27.5% of participants exhibited a significantly higher likelihood of *E. coli* detection (OR = 21.002; *P* = 0.036), whereas no significant association was detected for *Salmonella* spp. (**Figure 2**). Additionally, recent antibiotic use (within the past 3 months) among 23 participants was linked to higher prevalence of both *E. coli* (47.83% versus (vs.) 23.53%) and *Salmonella* spp. (8.69% vs. 0%) compared to non- users, although these differences were not statistically significant.

**Figure 3:**
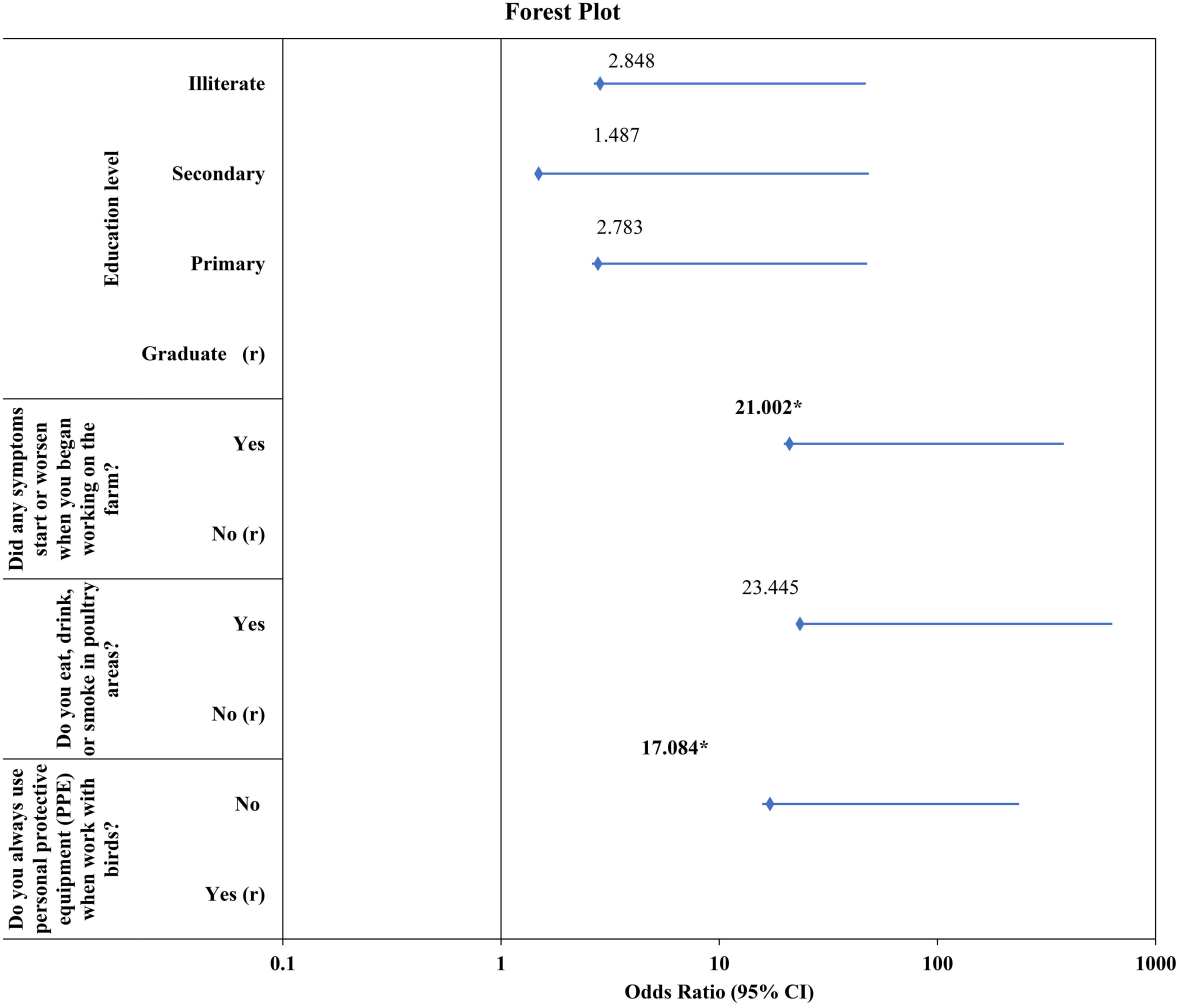
Forest plot illustrates the multivariate logistic regression analysis of independent risk factors associated with *E. coli* isolation in the examined farms. Data presented as odds ratios with 95% confidence intervals [OR (95% CI)]. *Statistically significant at *P* < 0.05.

Focusing hygiene knowledge and practices, hand-washing frequency showed a strong and significant association with *E. coli* isolation (*P* < 0.05). Workers who washed their hands only when visibly dirty had the highest positivity rate (100%), followed by those who washed only at the end of the workday (80%). Workers, who ate, drank, or smoked in poultry areas showed substantially a significantly increased the risk of *E. coli* isolation, being 36 times more likely to be positive than withthose who did not behave (*P* < 0.05). The use of personal protective equipment (PPE) during bird handling was associated with a significantly lower rate of *E. coli* positivity, whereas failure to use PPE was associated with a 17.08-fold higher risk of *E. coli* isolation (**Figure 3**; **Supplementary Table 2**). Additionally, failure to change PPE especially boots or gloves between poultry houses was significantly associated with *E. coli* positivity (43.8% vs. 7.7%; *P* = 0.031), as shown in **Table 3**.

### 3.4 Antimicrobial susceptibility of *E. coli* and *Salmonella* spp.

**Figure 4:**
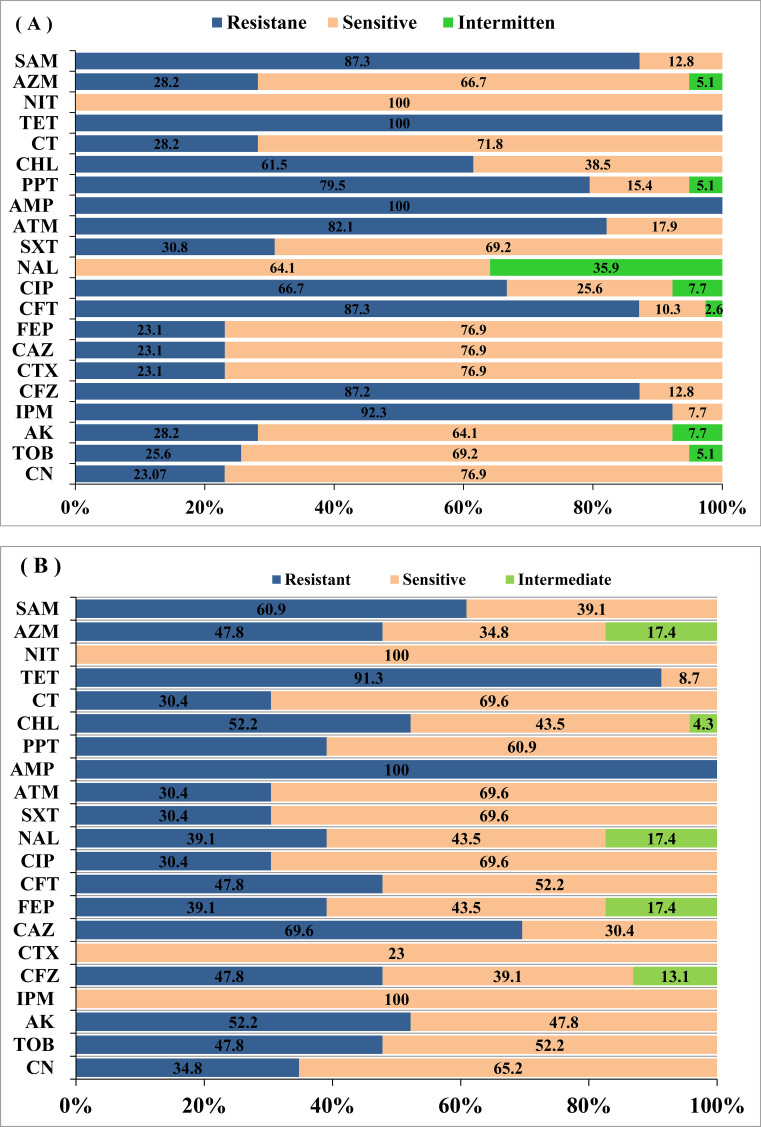
Stacked bar chart showing antimicrobial resistance of **(A)** *E. coli* (%) and **(B)** *Salmonella* spp. in different sources in examined poultry farms markets.

The phenotypic resistance patterns of 39 *E. coli* and 23 *Salmonella* spp. isolates of the 21 antibiotics belonging to fifteen different classes determined via the Kirby–Bauer disk diffusion method are shown in **Supplementary Table 3**. All *E. coli* isolates (100%) and most *Salmonella* spp. isolates (91.3%) presented resistance patterns, as exhibited in **Figure 4** and **Supplementary Table 3**. *E. coli* isolates exhibited full resistance to AMP and TET (100%) and an elevated degree of resistance to IPM (92.3%), CFT (87.3%), SAM (87.3%), CFZ (87.2%), ATM (82.1%), and PPT (79.5%). However, the isolates demonstrated full susceptibility to NIT (100%). *Salmonella* spp. showed full resistance to AMP (100%), a relatively high degree of resistance to TET (91.3%), and 100% susceptibility to IPM and NIT, as exhibited in **Figure 4** and **Supplementary Table 3**. The MAR indices ranged from 0.24 to 0.9, with an average of 0.57 for the *E. coli* isolates, and 0.09 to 0.76, with an average of 0.425 for the *Salmonella* spp. isolates (**Table 4**). Among the 39 examined *E. coli* isolates, 71.8% were MDR and 28.2% were XDR, as detailed in **Table 3**. In addition, 60.9% of the 23 *Salmonella* spp. isolates examined were MDR, and 30.4% were XDR, as detailed in **Table 4**.

**Table 4:** Distribution of virulent genes and antibiotic resistance patterns in *E. coli* and *Salmonella* spp. isolates.

Serotypes	Source	Number of isolates	Virulence genes patterns	Antibiotic resistance patterns	MAR index
E.coli (n=39)					
*E.coli* O111:H2	Broiler	9	*stx*1, *stx*2, *eae*A, *hyl*A	CN, TOB, AK, PPT, IPM, CFZ, CTX, CAZ, FEP, CFT, CIP, SXT, ATM, AMP, SAM, CHL, CT, AZM, TET^XDR^	0.9
*E.coli* O26:H11	Broiler	2	*stx*1, *stx*2, *hyl*A	AK, IPM, CFZ, CFT, CIP, ATM, AMP, SAM, CHL, CT, AZM, TET^XDR^	0.57
*E.coli* O1:H7	Feedstuff	1	*eae*A, *hyl*A	TOB, PPT, IPM, CFZ, CFT, ATM, AMP, SAM, CHL, TET^MDR^	0.48
*E.coli* O128:H2	Human	3	*stx*1, *stx*2, *hyl*A	PPT, IPM, CFZ, CFT, CIP, SXT, ATM, AMP, SAM, TET^MDR^	0.48
*E.coli* O127:H6	Broiler	4	*stx*1, *stx*2, *hyl*A	PPT, IPM, CFZ, CFT, CIP, ATM, AMP, SAM, TET^MDR^	0.43
*E.coli* O17:H8	Litter	1	*eae*A, *hyl*A	PPT, IPM, CFZ, CFT, CIP, ATM, AMP, SAM, TET^MDR^	0.43
*E.coli* O125:H21	Drinkers	3	*stx*1, *hyl*A	PPT, IPM, CFZ, CFT, CIP, ATM, AMP, SAM, TET^MDR^	0.43
*E.coli* O55:H7	Broiler	2	*hyl*A	PPT, IPM, CFZ, CFT, CIP, ATM, AMP, SAM, TET^MDR^	0.43
*E.coli* O44:H18	Drinkers	2	*eae*A, *hyl*A	PPT, IPM, CFZ, CFT, CIP, AMP, SAM, CHL, TET^MDR^	0.43
*E.coli* O112:H21	Litter	2	*stx*1, *stx*2, *eae*A	PPT, IPM, CFZ, CFT, AMP, SAM, CHL, TET^MDR^	0.38
*E.coli* O121:H7	Drinkers	1	*eae*A	IPM, CFZ, CFT, ATM, AMP, CHL, TET^MDR^	0.33
*E.coli* O15:H2	Human	1	*eae*A	PPT, IPM, CFZ, AMP, SAM, CHL, TET^MDR^	0.33
*E.coli* O103:H2	Feedstuff	1	*stx*1, *stx*2, *eae*A	PPT, IPM, ATM, AMP, SAM, CHL, TET^MDR^	0.33
*E.coli* O146:H21	Drinkers	1	*eae*A	PPT, IPM, ATM, AMP, SAM, CHL, TET^MDR^	0.33
*E.coli* O111:H4	Human	1	*stx*2, *hyl*A	PPT, IPM, ATM, AMP, SAM, TET^MDR^	0.29
*E.coli* O114:H4	Drinkers	1	*eae*A	IPM, CFT, ATM, AMP, SAM, TET^MDR^	0.29
*E.coli* O79:H7	Litter	1	*stx*1, *stx*2	IPM, CFT, ATM, AMP, CHL, TET^MDR^	0.29
*E.coli* O113:H4	Drinkers	1	*stx*1, *stx*2	CFZ, CFT, AMP, CHL, TET^MDR^	0.24
*E.coli* O1:H7	Human	1	*hyl*A	CFZ, CFT, AMP, CHL, TET^MDR^	0.24
*E.coli* O91:H21	Drinkers	1	*stx*1, *stx*2	CFZ, ATM, AMP, CHL, TET^MDR^	0.24
*Salmonella* spp. (n=23)					
*S. enteritidis*	Broiler	1	*sop*B, *stn*, *hil*A, *spv*C	CN, TOB, AK, PPT, FEP, CFZ, CFT, CIP, SXT, ATM, AMP, SAM, CHL, CT, TET, AZM^XDR^	0.76
*S. enteritidis*	Broiler	4	*sop*B, *stn*, *hil*A, *spv*C	CN, TOB, AK, PPT, CFZ, CAZ, CFT, CIP, SXT, ATM, AMP, SAM, CHL, CT, TET, AZM^XDR^	0.76
	Human	1			
	Drinkers	1			
*S. Kentukey*	Broiler	2	*sop*B, *stn*, *hil*A	TOB, AK, ATM, AMP, CAZ, CHL, TET, AZM^MDR^	0.38
*S. typhimurium*	Broiler	2	*sop*B, *stn*, *hil*A	CFZ, CAZ, FEP, NAL, AMP, SAM, TET^MDR^	0.33
*S. typhimurium*	Litter	1	*sop*B, *stn*, *hil*A	CFZ, CAZ, FEP, NAL, AMP, SAM, TET^MDR^	0.33
*S. typhimurium*	Human	1	*sop*B, *stn*, *hil*A	CFZ, CAZ, FEP, NAL, AMP, SAM, TET^MDR^	0.33
*S. Wingrove*	Broiler	3	*sop*B, *stn*, *hil*A	CAZ, FEP, ATM, AMP, SAM, CHL, TET^MDR^	0.33
*S. Bargny*	Broiler	1	*sop*B, *stn*, *hil*A	CAZ, FEP, NAL, AMP, SAM, TET^MDR^	0.29
*S. anatum*	Broiler	3	*sop*B, *hil*A, *spv*C	CFT, NAL, AMP, TET^MDR^	0.19
*S. anatum*	Litter	1	*sop*B, *hil*A, *spv*C	CFT, NAL, AMP, TET^MDR^	0.19
*S. Virchow*	Broiler	2	*sop*B, *stn*, *hil*A	PPT, AMP	0.09

CN, gentamicin; TOB, tobramycin; AK, amikacin; PPT, piperacillin-tazobactam; IPM, imipenem; CFZ, cefazolin; CTX, cefotaxime; CAZ, ceftazidime; FEP, cefepime; CFT; cefoxitin; CIP, ciprofloxacin; NAL, nalidixic acid; SXT, trimethoprim-sulphamethoxazole; ATM, aztreonam; AMP; ampicillin; SAM, ampicillin–clavulanic acid; CHL, chloramphenicol; CT, colistin; TET; tetracycline; NIT, nitrofurantoin; AZM, azithromycin, 1 MDR, multiple drug resistant; XDR, extensive drug resistant; MAR, multiple antibiotic index.

### 3.5 Virutyping of *E. coli* and *Salmonella* spp. isolates

The 16S *rRNA* gene was molecularly identified in biochemically suspected 39 *E. coli* and 23 *Salmonella* spp. Four virulence-associated genes were selected for identification in each of the 39 *E. coli* isolates (*stx*1, *stx*2, *eae*A, and *hyl*A) and each of the 23 *Salmonella* spp. isolates (*sop*B, *stn*, *hil*A, and *spv*C) (**Table 4**). The *stx*1 and *stx*2 virulence genes were detected in 69.2% of the examined *E. coli* isolates in this study; in addition, a high prevalence of the *hyl*A virulence gene (74.4%) and a lower prevalence of the *eae*A virulence gene (48.7%) were detected (**Table 4**). The *sop*B and *hil*A genes were identified in all examined *Salmonella* spp. isolates (100%), and most of the *stn* virulence genes were identified in *Salmonella* spp. isolates (82.6%), whereas the *spv*C gene had a low prevalence (43.5%).

### 3.6 Antimicrobial activity of natural melanin

The disk diffusion method was used to evaluate the antimicrobial effects of melanin derivatives from *A. flavus* on *E. coli* and *S. enteritidis* counts. The inhibition zone diameters of the *E. coli* and *S. enteritidis* isolates treated with prepared melanin were 23 mm and 21 mm, respectively. The growth of melanin-treated *E. coli* and *S. enteritidis* declined at 24 h and reached complete inhibition at 120 h of incubation. However, the control plates showed an increase in growth of non-treated *E. coli* and *S. enteritidis*as presented in **Supplementary Table 4**. The MICs for melanin-treated *E. coli* and *S. enteritidis* isolates in this study were 0.3 mg/mL and 0.2 mg/mL, respectively upon using the tube dilution method, Whereas, the corresponding MBC values were 0.6 mg/mL and 0.4 mg/mL for the melanin-treated *E. coli* and *S. enteritidis* isolates.

### 3.7 Anti-virulent activity of natural melanin

**Figure 5:**
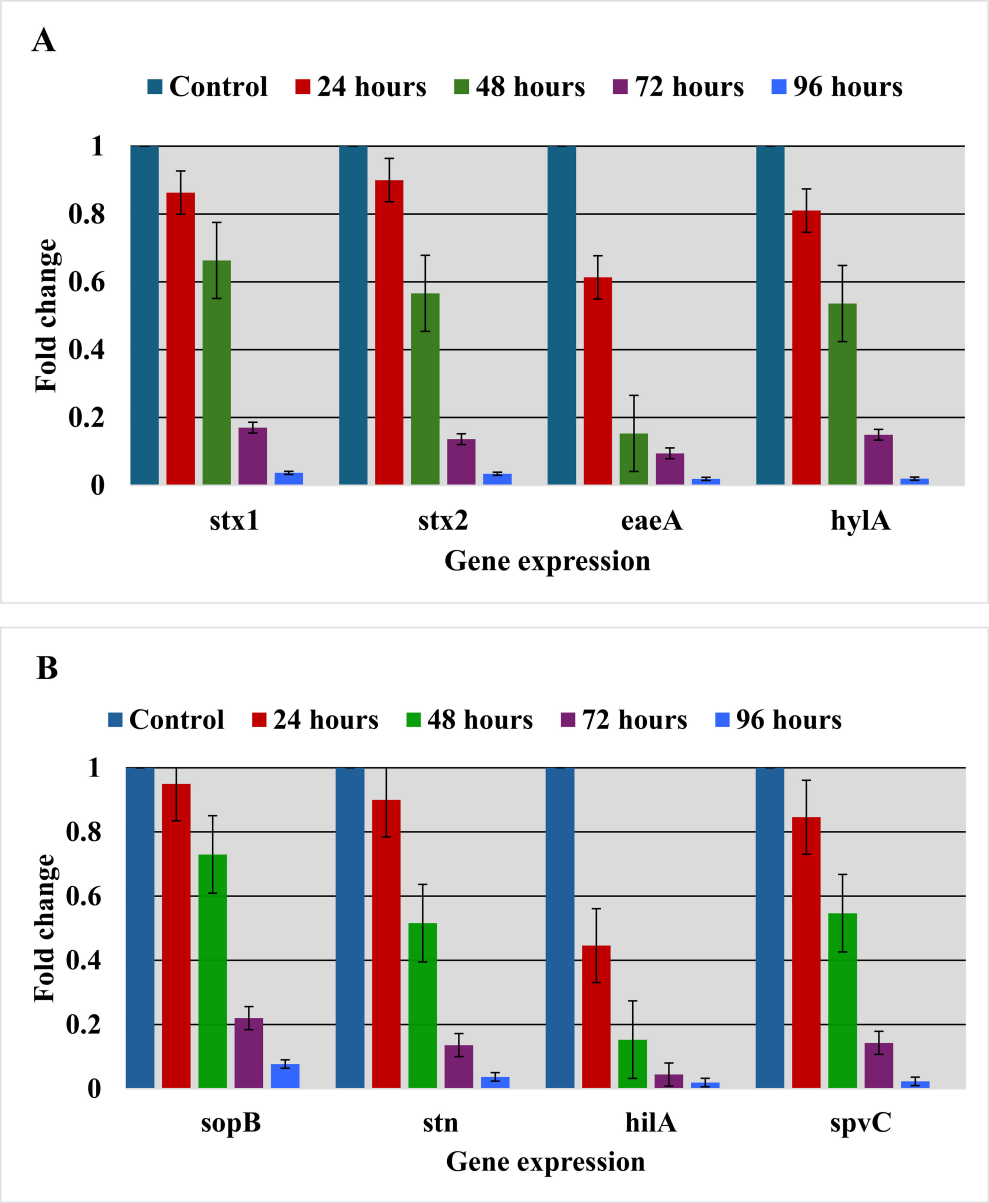
The relative m*RNA* expression levels of **(A)** *E. coli* and **(B)** *S. enteritidis* associated virulence genes before and after treatment with natural melanin.

The expression of virulence genes of melanin-treated *E. coli* isolates (*stx*1, *stx*2, *eae*A, and *hyl*A) and *S. enteritidis* isolates (*sop*B, *stn*, *hil*A, and *spv*C) at time intervals of 24, 48, 72, 96, and 120 h is displayed (**Figure 5**). The expression levels of virulence genes significantly decreased over the incubation periods, and reached a complete reduction at 120 h in both the *E. coli* and the *S. enteritidis* isolates in this study. As shown in **Supplementary Table 5**, there were significant differences in the relative mRNA expression levels of *E. coli*- and *S. enteritidis*- associated virulence genes after treatment with natural melanin across the incubation periods.

## 4 Discussion

The presence of *E. coli* in broilers is considered a potential public health concern due to its ability to be transmitted from poultry to humans (Laube et al., 2014), which may complicate the treatment of human infections (Lazarus et al., 2015). In the present study, the overall prevalence of *E. coli* was 51.75%, which is similar to earlier findings from Egypt (Zakaria et al., 2018) and China (Zhou et al., 2024). Even so, our results were lower than those reported from Egyptian broiler farms, where the prevalence reached 79.76% (Zaki et al., 2024). Conversely, the percentage we recorded was higher than those previously reported in Egypt (Kaoud et al., 2020) and in Romania (Bratfelan et al., 2023).

Among our samples, cloacal swabs had the highest detection rate of *E. coli* (61.67%), followed by drinking water (54.44%), litter (44.44%), workers’ hand swabs (37.5%), and feed (22%). These values exceed those recorded by Kaoud et al. (2020), who reported *E. coli* in 27% of cloacal swabs, and only 7% of litter samples in Egypt. A lower prevalence was also noted in Lithuania, where Kasparaviciene et al. (2025) reported overall rates of 29.17% of cloacal swabs, and 13.88% for farm environmental samples. Similarly, Saha et al. (2020) reported lower levels in Bangladesh, with 17.82% in cloacae swabs, and even lower rates in feed (5.17%), water (9.20%), and workers’ hand swabs (10.34%). In contrast, several studies have reported higher prevalence rates. Zhou et al. (2024) reported 91.4% of cloacal swabs, followed by 68.6% in poultry workers’ hand swabs, and 35.7% in feed in China. Zaki et al. (2024) reported widespread contamination of *E. coli* in cloacal swabs (100%), drinking water (91.6%), litter (91.6%), feeders (83.3%), and workers’ hand swabs (58.3%). In another investigation, high levels were also recorded among broiler cloacal swabs (71.05%) in the Mekong Delta, Vietnam (Nguyen et al., 2021), as well as among poultry workers themselves (Aworh et al., 2021).

The high proportion of *E. coli* detected in cloacal swabs in our study is consistent with the natural habitat of *Enterobacteriaceae* in the avian intestine. Thus, the intestinal tract of poultry serves as a primaryand continuous source of *E. coli* contamination in the farm environment (Nguyen et al., 2022). Consequently, the presence of *E. coli* in litter, water feed and other farm components reflects ongoing fecal contamination and an active transmission cycle between birds and humans (Shtylla et al., 2023).

Hygiene plays a central role in controlling *E. coli*, as this bacterium is recognized as a major contributor to poor flock performance, early mortality, and significant economic losses due to colibacillosis (Fancher et al., 2020). Inadequate sanitation, the accumulation of fecal material and improper water management create favorable conditions for the persistence of *E. coli* (Nguyen et al., 2022). Water sources can become contaminated with fecal matter, promoting infection among birds that drink this water (Feng et al., 2023**;** Ngwenya et al., 2023). Marinating clean water systems and robust biosecurity programs, including restricted access to poultry houses, effective disinfection routines, the use of transition zones, and pest control, can significantly limit the spread of *E. coli* (Tilli et al., 2022).

*Salmonella* spp. were detected in 5.75% of all the examined samples. This prevalence is lower than that reported in several countries, including Egypt (10.37%, Soliman et al., 2018; 10.7%, Zaki et al., 2023**;** 17%, Elsayed et al., 2024), Ethiopia (14.4%, Waktole et al., 2024), Iran (36.4%, Ansari-Lari et al., 2022) and Algeria (34.37%, Djeffal et al., 2018). Our results were higher than those reported in Denmark (1.8%, DTU Findit, 2018) and Poland (1.57%, Witkowska et al., 2021).

The distribution of *Salmonella* spp. across sample types varied with cloacal swabs showing the highest positivity (10%), followed by poultry workers’ hand swabs (5%) and litter (3.7%). Only, 1.1% drinker water was positive, while, no *Salmonella* spp. were recovered from the feed samples. These findings are markedly lower than those of earlier Egyptian reports, by Zaki et al. (2023), where the cloacal swabs, litter, and feed were 41.7%, 25% and 8.3%, respectively. Higher rates have also been reported in Ethiopian broiler farms, where litter (18.54%), and poultry workers’ hand swabs (18.45%) were highly contaminated, followed by cloacal swabs (12.90%), and feedstuff (7.89%) (Basazinew et al., 2025). Other Ethiopian studies reported even higher recovery rates in litter (19.7%) and cloacal swabs (14.5%), while similar levels were noted in feed and water (8.5%, each) samples (Waktole et al., 2024). In comparison, studies from central Ethiopia reported much lower isolation rates, ranging from 0.3% in cloacal swabs to 3.4% in litter (Dagnew et al., 2020**).** These findings highlight the roles of both human activity and environmental contamination in *Salmonella* spp. dissemination (Basazinew et al., 2025).

Poor hygiene and farm management practices play fundamental roles in *Salmonella* spp. persistence. Common risk factors include improper handling of farm waste, contamination of feed with fecal matter, insufficient cleaning and disinfection, rodent infestation and cross contamination along the poultry production chain. Human behavior, such as inadequate hand hygiene and failure to use protective gloves when handling chickens, further increases the risk of *Salmonella* spp. transmission (Abdi et al., 2017**;** Odoch et al., 2017**;** Eguale, 2018**;** Taddese et al., 2019**;** Dagnew et al., 2020**;** Jibril et al., 2020). To minimize these risk factors, thorough cleaning of equipment and sanitization between flocks, along with comprehensive biosecurity training for farm workers are essential (Tilli et al., 2022).

Among the *E. coli* isolates, ninety different serotypes were identified, serotypes O111:H2, and O127:H6 were the predominant serotypes, with percentages of 25.64%, and 10.25%, respectively, in this study, which is consistent with previous studies by Daoud et al. (2016) and Nemati et al. (2025). Seven different *Salmonella* serotypes were identified, with the highest predominance of *S. enteritidis*, *S. typhimurium*, and *S. anatum* in this study. *S. enteritidis* and *S. typhimurium* were the predominant species isolated from poultry farms in previous reports in Egypt (Elkenany et al., 2019**;** Abou Elez et al., 2021) and Brazil (Borges et al., 2013).

Understanding how risk factors influence the prevalence of bacterial isolates has a crucial role in preventing cross-contamination in poultry farms. In this study, most socio-demographic variables were not significant associations with the isolation of *E. coli* or *Salmonella* spp. isolation among poultry workers. This aligns with reports from Nigeria and Ethiopia, where *E.coli* and *Salmonella* spp. did not differ by age or gender (Aworh et al., 2019; Taddese et al., 2019). The key reason reflect the nature of occupational zoonotic exposure, where pathogens can disperse throughout poultry environments via aerosols, feces and contaminated surfaces, resulting in relatively exposure risks regardless of personal characteristics (Aworh et al., 2019; Mpundu et al., 2019).

Education level showed a borderline association (*P*=0.053) with bacterial isolation, suggesting that education may help workers in translating knowledge into protective behaviors.Higher education levels may facilitate better recognition of biosecurity risks and adherence to preventive practices, whereas lower education may impair risk recognition and contribute to lapses in hand washing, waste handling and other biosecurity controlled measures. In our study, educated workers showed lower *E. coli* prevalence (12.5%) and appeared more likely to adopt measures such as disinfection and bird quarantine Evidence from Nigeria and global studies supported that, lower-education groups show higher prevalence of MDR *E. coli* (OR = 2.1; Aworh et al., 2019) and elevated foodborne *E. coli* risk (OR=1.8; Pinto et al., 2023), which is consistent with reduced access to wash training and weaker implementation of biosecurity practices.

Only 22.5% of the participants demonstrated basic biosecurity knowledge, while 60% understood the routes of disease transmission, both groups showed lower pathogen positivity with no statistical significance differences. This finding is consistent with the conclusions of Aworh et al. (2019), who identified biosecurity knowledge including hygiene protocols and transmission mechanisms as critical determinants in preventing pathogen entrance and dissemination in poultry farms. Low biosecurity awareness may facilitate colonization and spread of pathogens as *E. coli* through the fecal-oral route, particularly in environments with inadequate sanitation and limited knowledge of transmission and contamination risks (Aworh et al., 2021). Concerning, Pre-existing health conditions the highest *E. coli* isolation was detected in participants with multiple symptoms, while *Salmonella* spp. positivity was concentrated among those with gastrointestinal complaints. Our results parallel the data from Nigeria in 2019, where a higher prevalence of *E. coli* was reported among symptomatic patients particularly those with diarrhea (OR = 3.3), which was attributed to a disrupted gut microbiota that facilitate colonization by MDR strains. Immunological impairment and compromised epithelial integrity associated with pre-existing health conditions enhance the host susceptibility to infection by promoting pathogen adhesion, toxin damage and potential systemic spread (Aworh et al., 2021). The strong association between symptom worsening after beginning farm work and *E. coli* occurrence may reflects frequent occupational exposure to pathogenic strains. Such exposures can lead to intestinal colonization and development of acute symptoms, includingdiarrhea, highlighting occupational contact as a major transmission route (Rahman et al., 2017; Aworh et al., 2019). In contrast, the absence of a similar pattern for *Salmonella* may stem from differences in its ecology and transmission behavior (Economou and Gousia, 2015). Participants with previous antibiotic use showed a notable increase in pathogen positivity. This observation is aligned with the broad depletion of normal microbiota caused by antibiotics, which lowers microbial diversity and allows resistant *Enterobacteriaceae* to proliferate (Zhang and Chen, 2019). Furthermore, *Salmonella* prevalence aligns with co-selection mechanisms, in which farm biocides are linked to resistance genes, increasing pathogen fitness and promoting horizontal gene transfer that enhances their persistence and dissemination (Wales and Davies, 2015**).**

In terms of hygienic practices, workers who washed their hands only when they are visibly dirty have the highest pathogen positivity, since microscopic pathogens can persist unnoticed without visible soiling and accumulate, increasing cross-transmission risk (Allegranzi and Pittet, 2009**;** Kampf et al., 2009). Engaging in high-risk behaviors as eating, drinking, or smoking within poultry farming areas are associated with a greatly increased risk of *E. coli* infection by 36-fold compared to workers who avoid these practices. This agreed with Abebe et al. (2020), who highlights the importance of hand-to-mouth transmission in acquiring zoonotic pathogens. Because poultry environments are often heavily contaminated with pathogenic *E. coli*, these behaviors bypass natural protective barriers and allow direct ingestion of bacteria from hands, aerosols or surfaces and promoting intestinal colonization (Mughini Gras et al., 2019). Proper use of PPE plays a vital role in lowering *E. coli* positivity among participants. Poultry workers, who did not use PPE had a 17.08-fold higher risk of *E. coli* isolation. Changing or cleaning PPE between poultry houses also greatly substantially reduced positivity from 43.8% to 7.7%, underscoring its role in preventing cross-house transmission. These findings align with studies showing that PPE acts as a barrier against fecal contamination in poultry environments, reducing microbial loads and zoonotic dissemination (Mughini Gras et al., 2019**;** Abebe et al., 2020). In the absence of PPE, workers may have exposed to contaminated materials, facilitating pathogen transfer, persist and accumulate explaining the markedly higher isolation rates.

The prolonged use of antimicrobial agents for the treatment of *E. coli and Salmonella* spp. infection in broiler farms results in the generation of multidrug-resistant strains that transfer antimicrobial resistance genes to other bacteria in human intestinal microflora, resulting in acquired zoonoses (Schroeder et al., 2003**;** Durrani et al., 2024**;** Oubouyahia et al., 2025). The *E. coli* and *Salmonella* spp. isolates in this study exhibited full resistance (100%) to AMP and TET. On the other hand, a study by Khong et al. (2023) reported the lowest resistance of *E. coli* to ampicillin, tetracycline, and colistin. AMP and TET were not effective against *E. coli* and *Salmonella* spp. isolates in the present study, which is consistent with the findings of Singh et al. (2013) in India, Andoh et al. (2016) in Ghana, Abou Elez et al. (2021) in Egypt, Mooljuntee et al. (2010) in Thailand, and Shang et al. (2023) in Korea. Many studies have highlighted high rates of resistance in *E. coli* and *Salmonella* spp. to tetracycline, amoxicillin, and ciprofloxacin (Ssemakadde et al., 2025**;** Ranasinghe et al, 2022**;** Miles et al., 2006). Tetracycline resistance is regulated by efflux genes that are associated with large plasmids that are transformed from a sensitive strain and are conjugated with other bacterial resistance genes or toxins in gram-negative bacteria (Speer et al., 1992). The excessive use of antimicrobials as a growth promoter and treatment for infection in livestock farming is considered the main cause of the high resistance frequency and emergence of resistant bacteria in animals and humans in contact (Brown et al., 2017). The high resistance of *E. coli* isolates to a variety of antibiotics, such as IPM, CFT, SAM, CFZ, ATM, and PPT, in this study was inconsistent with that reported by Khong et al. (2023), who reported the lowest resistance to imipenem (1.5%). The *E. coli* isolates were fully sensitive to NIT (100%) in this study, suggesting the use of NIT in the treatment of *E. coli* infection. The susceptibility rates of *E. coli* to NIT reported in previous studies were 83% in India (Mamani et al., 2015), 88% in Morocco (Shariff et al., 2013), 94.1% in Iran (El Bounamri et al., 2014), 97.6% in Tunisia (Daoud et al., 2016), and 96% to 99% in western countries (Schito et al., 2009**;** Passadouro et al., 2014). All *Salmonella* isolates in this study were sensitive to IPM and NIT, which was attributed to the limited use of these antimicrobials in commercial chicken farms (Abou Elez et al., 2021). Carbapenem antibiotics are recommended as suitable antibiotics for treating MDR bacterial infections, which are resistant to other classes of antibiotics (Aurilio et al., 2022). Drug-resistant *E. coli* isolates and *Salmonella* isolates accounted for 100% and 93.2% of the isolates in this study, respectively (**Table 2**), with average MAR values above 0.2, indicating that multidrug-resistant strains, which have been reported in *Salmonella* (Abdeen et al., 2018**;** Sharma et al., 2019) and *E. coli* (Afayibo et al., 2022**;** Ranasinghe et al., 2022**;** Ibrahim et al., 2023**;** Rafiq et al., 2024), make these antimicrobials ineffective in the treatment of human and poultry bacterial infections. This contradiction with our results could be due to the method used, geographical area, sample type, and sample size. Among the tested *E. coli* isolates, 71.8% exhibited MDR, and 28.2% exhibited XDR in this study. On the other hand, 88.9% of *E. coli* isolates from chickens were XDR, and 11.1% were MDR in Egypt (Ezzat et al., 2023). However, a study by Monroy et al. (2025) reported that 100% of *E. coli* isolates were MDR resistant and that 23.3% were XDR. *Salmonella* spp. were MDR in 60.9% and XDR in 30.4% of the examined isolates in this study, which was higher than the percentage of MDR *Salmonella* isolated from chickens in the USA (Alali et al., 2010) and China (Bai et al., 2015**;** Zhao et al., 2016). However, the prevalence of MDR *Salmonella* spp. in the present study was lower than the 100% reported previously in chickens in Egypt (Abdeen et al., 2018).

The diversity of virulence factors in *E. coli* and *Salmonella* spp. causes their adaptation, survival, and pathogenicity in the host (Elemfareji and Thong, 2013**;** Mansour et al., 2023). The ability of Shiga toxin-producing *E. coli* (STEC) to cause severe human illness is associated with the production of two powerful cytotoxins, *stx*1 and *stx*2, which inhibit the synthesis of proteins in the host cell, leading to cell death (Melton-Celsa, 2014). The *hyl* and *eae*A genes are involved in the virulence of *E. coli* (Emery et al., 1992). Intimin is an external protein response that allows bacteria to attach to intestinal host cells, causing cell damage (Exeni et al., 2018). Enterohemolysin, encoded by the *hyl*A gene, plays a role in the pathogenicity of STEC, liberates hemoglobin from RBCs, and causes cell damage (Smith and Fratamico, 2017). Therefore, the detection of the *stx*1, *stx*2, *eae*A, and *hyl*A genes was important in this study. The *stx*1 and *stx*2 genes were detected in 69.2% of the tested *E. coli* isolates in this study; the *hly*A gene was detected in 74.4%, whereas *eae*A was detected in 48.7% in this study. A study by Mansour et al. (2023) revealed the presence of each *stx*1 and *stx*2 gene in 23.8%, the *eae*A gene in 90.5% and the *hly*A gene in 71.4% of Egypt. However, previous studies by Wani et al. (2004); Schroeder et al. (2003), and Jamshidi et al. (2016) could not isolate *stx*1 and *stx*2 in chickens.

The *sop*B and *hil*A virulence genes play a role in *Salmonella* invasion of host cells (Huehn et al., 2010). However, the enterotoxin *Salmonella*-associated *stn* gene is responsible for toxin production and diarrhea in the host (Huehn et al., 2010), and the *spv* gene aids in the survival and replication of *Salmonella* spp. in the host cell (Fluit, 2005). The *sop*B and *hil*A genes were identified in all the *Salmonella* spp. isolates examined in this study, followed by the stn virulence gene (82.6%), in alignment with a previous study in Malaysia (Thung et al., 2018). In contrast to Thung et al. (2018), who could not detect the *spv*C gene in *Salmonella* spp. isolates, 43.5% of the *spv*C gene was detected in this study.

Gram-negative bacteria are difficult to treat because of the presence of an additional membrane layer in their cell walls, which prevents the penetration of antibiotics deeper into bacterial cells (Xu et al., 2017). A variety of fungi produce melanin pigments under submerged fermentation conditions (Aghajanyan et al., 2005). The broad-spectrum actions of different melanins can inhibit a variety of Gram-positive bacteria and Gram-negative bacteria (Ali and Haq, 2010**;** Wang et al., 2019). Melanin is a negatively charged, hydrophobic pigment of high molecular weight composed of multifunctional polymers and polyphenolic compounds; these phenolic and indolic compounds are known for their antimicrobial activity (Pal et al., 2014). Melanin damages the integrity of both Gram-negative bacteria and Gram-positive membranes and causes cell leakage (Xu et al., 2017). Thus, the application of natural pigments such as melanin could be an alternative way to overcome multidrug-resistant bacteria in recent years (Mao-Sun et al., 2007**;** Rani et al., 2013), especially *E. coli* and *Salmonella* spp. (Vasanthabharathi et al., 2011**;** Rani et al., 2013). The presence of a clear zone around the melanin disc in inoculated bacterial plates in our study suggests strong antimicrobial activity of natural melanin derivatives from *A. flavus* against drug-resistant bacteria, causing a reduction in bacterial number (Mohagheghpour et al., 2000**;** Rani et al., 2013). The zones of inhibition of melanin-treated *E. coli* and *S. enteritidis* were 23.3 ± 0.3 mm and 21.6 ± 0.7 mm, respectively, in this study, in accordance with the findings of Mohagheghpour et al. (2000), who reported an inhibition zone of 20 mm for *E. coli* treated with melanin. Vasanthabharathi et al. (2011) reported inhibition zones of 18 mm for *E. coli*, 12 mm for *S. typhi*, and 11 mm for *S. paratyphi*. Salomi et al. (2023) reported that the maximum activity was recorded against *E. coli* (32* mm*), followed by *S. typhi* (23* mm*). The MIC and MBC values were determined in our study, revealing the extent of variation with Xu et al. (2017), who reported that the MIC values of *E. coli* and *S. typhi* were 0.4 mg/mL and 2.4 mg/mL, respectively, and the MBC values of *E. coli* and *S. typhi* were 0.4 mg/mL and 3.2 mg/mL, respectively. While, the MICs values of melanin derived from *Streptomyces djakatensis* were 6.25 µg/mL and 25 µg/mL against *Pseudomonas aeruginosa* and *Staphylococcus aureus*, respectively (El-Zawawy et al., 2024). The least antimicrobial activity of melanin against *E. coli* (El-Zawawy et al., 2024). In contrast, Elsayis et al. (2022), and Kumar et al. (2011) demonstrated that the melanin had no antimicrobial activity against *E. coli*.

The melanin pigment exhibited variable antimicrobial activity against different bacterial spp., with different inhibitory zones in diameter depending on the bacterial spp., the amount, source, and concentration of melanin (Laxmi et al., 2016**;** Xu et al., 2017**;** El-Zawawy et al., 2024). The diameter of inhibition zones, and the antimicrobial activity of melanin rised when the amount of melanin increased in this study.

In addition to exploring the effects of natural melanin derivatives from *A. flavus* on the growth of *E. coli* and *S. enteritidis* in this study, we examined their effects on the expression of virulence genes in both bacteria. The counts of the melanin treated *E. coli* and *S. enteritidis* isolates decreased after different incubation times, and growth was completely absent after 120 h of incubation in this study. Le et al. (2024) reported that melanin derivatives from *Fomes fomentarius* and *Daedaleopsis tricolor* samples did not significantly inhibit bacterial growth. The gene expression experiment showed that melanin significantly decreased the expression of *E. coli* and *S. enteritidis* virulence associated genes. Thus, melanin has the potential to target *E. coli* and *S. enteritidis* virulence genes in this study, suggesting that melanin could be a novel therapeutic approach for treating colibacilosis and salmonelosis.

## 5 Conclusion

In this study, the *E. coli* serotypes O111 and *S. enteritidis* were frequently prevalent serotypes isolated from broilers, water, and humans at different broiler farms in Sharkia Governorate, Egypt. The recovery of MDR and XDR strains of *E. coli and Salmonella* spp. indicates the improper use of antibiotics. NIT was considered the drug of choice in this study for the treatment of *E. coli* and *Salmonella* infection in broiler farms and humans. This study revealed that natural melanin derivatives from *A. flavus* have potential antimicrobial and anti-virulence activities against XDR *E. coli* and *S. enteritidis* and could be recommended for use on broiler farms. Further studies on the antimicrobial effects of melanin on other bacterial spp. are warranted.

## Data Availability

The original contributions presented in the study are included in the article/**Supplementary Material**. Further inquiries can be directed to the corresponding authors.
