# Supplementary material for: Antimicrobial and anti-virulent efficacies of melanin, and risk factors associated with Escherichia coli and Salmonella species from broiler farms and humans: integrating a one health approach
Source: Front Cell Infect Microbiol. 2026 Jul 20;16:1868544. doi: 10.3389/fcimb.2026.1868544 (PMC13429498; doi:10.3389/fcimb.2026.1868544)
Supplement: Supplementary file 1 [file Table1.docx]

Supplementary Material

**Antimicrobial and anti-virulent efficacies of melanin, and risk factors associated with *Escherichia coli* and *Salmonella* species from broiler farms and humans: Integrating a one health approach**

**Amira S. A. Attia^1*^, Nashwa El-Gazzar^2^, Hassan Mohmoud Diab^3^, Emad Sakr^4^, Rehab E Mohamed^5^, Ahmed Al-baqir^6^, Zeinab Saed Ibrahim^7^,Hend Abdalla El-sayed^7^, Ghada Abd Elmoniem Mokhtar^7^, Noura Almadani^8^, and Rasha M. M. Abou Elez^5*^**

^1^ Department of Veterinary Public Health, Faculty of Veterinary Medicine, Zagazig University, Egypt

^2^ Department of Botany and Microbiology, Faculty of Science, Zagazig University, Egypt

^3^ Department of Animal and Poultry Health and Environment, Faculty of Veterinary Medicine, Qena University, Egypt

^4^ Department of Animal Hygiene and Zoonoses, Faculty of Veterinary Medicine, University of Sadat city, Egypt

^5^ Department of Zoonoses, Faculty of Veterinary Medicine, Zagazig University, Egypt

^6^ Department of Avian and Rabbit Medicine, Faculty of Veterinary Medicine, Zagazig University, Egypt

^7^ Department of Medical Microbiology and Immunology, Faculty of Medicine, Zagazig University, Egypt

^8^ Community and Psychiatric Mental health nursing Department, College of Nursing, Princess Nourah bint Abdulrahman University, Riyadh 11671, Saudia Arabia

***Correspondence:**Rasha M.M. Abou Elez ([rmmohamed@zu.edu.eg](mailto:rmmohamed@zu.edu.eg))

Amira S. A. Attia ([dr.attiamirasamir@gmail.com](mailto:dr.attiamirasamir@gmail.com))

# Supplementary Tables

**Table Supplementary 1.** Nucleotide sequences and product sizes of *E. coli* and *S. enteritidis* primers.

| **Target gene** | **Primers sequences 5`-3`** | **Amplified segment** | **Primary**  **denaturation** | **Amplification (35 cycles)** | | | | **References** |
| --- | --- | --- | --- | --- | --- | --- | --- | --- |
|  |  |  |  | **Secondary denaturation** | **Annealing** | **Extension** | **Final extension** |  |
| *inv*A | F:GTGAAATTATCGCCACGTTCGGGCAA | 284 bp | 94°C (5 min.) | 94°C (30 sec.) | 55°C (30 sec.) | 72°C (30 sec.) | 72°C (7 min.) | Oliveira et al. (2003) |
|  | R:TCATCGCACCGTCAAAGGAACC |  |  |  |  |  |  |  |
| 16s *rRNA* | F:GAC CTC GGT TTA GTT CAC AGA | 585 bp | 95°C (3 min.) | 95°C (45 sec.) | 62°C (45 sec.) | 72°C (45 sec.) | 72°C (7 min.) | Seidavi et al. (2010) |
|  | R:CAC ACG CTG ACG CTG ACC A |  |  |  |  |  |  |  |
| *sopB* | F:TCAGAAGRCGTCTAACCACTC | 517 bp | 94°C (5 min.) | 94°C (30–40 sec.) | 58°C (40 sec.) | 72°C (45 sec.-1 min.) | 72°C (10 min.) | Huehn et al. (2010) |
|  | R:TACCGTCCTCATGCACACTC |  |  |  |  |  |  |  |
| *stn* | F:TTG TGT CGC TAT CAC TGG CAA CC | 617 bp | 94˚C (5 min.) | 94˚C (30 sec.) | 59˚C (40 sec.) | 72˚C  45 sec. | 72˚C (10 min.) | Murugkar et al. (2003) |
|  | R:ATT CGT AAC CCG CTC TCG TCC |  |  |  |  |  |  |  |
| *spvC* | F:ACCAGAGACATTGCCTTCC | 467 bp | 94°C (5 min.) | 94°C (30 sec.) | 55-58°C (30 sec.) | 72°C for 30-40s | 72°C (7 min.) | Huehn et al. (2010) |
|  | R:TTCTGATCGCCGCTATTCG |  |  |  |  |  |  |  |
| *hilA* | F:CATGGCTGGTCAGTTGGAG | 150 bp | 94˚C (5 min.) | 94˚C (30 sec.) | 60˚C (30 sec.) | 72˚C  30 sec | 72˚C (7 min.) | Yang et al. (2014) |
|  | R:CGTAATTCATCGCCTAAACG |  |  |  |  |  |  |  |
| *Stx1* | F:ACACTGGATGATCTCAGTGG | 614 bp | 94˚C (5 min.) | 94˚C (30 sec.) | 58˚C (40 sec.) | 72˚C 45 sec | 72˚C (10 min.) | Dipineto et al. (2006) |
|  | R:CTGAATCCCCCTCCATTATG |  |  |  |  |  |  |  |
| *Stx2* | F:CCATGACAACGGACAGCAGTT | 779 bp | 94˚C (5 min.) | 94˚C (30 sec.) | 58˚C (40 sec.) | 72˚C (45 sec.) | 72˚C (10 min.) |  |
|  | R:CCTGTCAACTGAGCAGCACTTTG |  |  |  |  |  |  |  |
| *eae*A | F:GTGGCGAATACTGGCGAGACT | 890 bp | 95 °C (3-5 min.) | 94 °C (30-60 sec.) | 55 °C - 64 °C (30-60 sec.) | 72 °C (30- 60 sec.) | 72 °C (5-7 min.) | Mazaheri et al. (2014) |
|  | R:CCCCATTCTTTTTCACCGTCG |  |  |  |  |  |  |  |
| *hyl*A | F:ACGATGTGGTTTATTCTGGA | 165 bp | 94°C (2 min.) | 94°C (20 sec.) | 60°C (1 min.) | 72°C (1 min.) | 72°C (7-10 min.) | Fratamico et al. (1995) |
|  | R:CTTCACGTGACCATACATAT |  |  |  |  |  |  |  |

| **Variables** | **OR** | **95% CI** | **P-value** |
| --- | --- | --- | --- |
| **Education level** | | | |
| - Illiterate | 2.848 | 0.18, 44.09 | 0.454 |
| - Primary | 2.783 | 0.17, 44.85 | 0.471 |
| - Secondary | 1.487 | 0.05, 47.03 | 0.822 |
| - Graduate | r |  |  |
| **Did any symptoms start or worsen when you began working on the farm?** | | | |
| - Yes | 21.002 | 1.23, 358.79 | **0.036*** |
| - No | r |  |  |
| **Do you eat, drink, or smoke in poultry areas?** | | | |
| - Yes | 23.445 | 0.90, 609.82 | 0.058 |
| - No | r |  |  |
| **Do you always use personal protective equipment (PPE) when work with birds?** | | | |
| - No | **17.08** | 1.33, 219.56 | **0.029*** |
| - Yes | r |  |  |

**Table S 2.** Multivariate logistic regression analysis of risk factors associated with *E. coli* isolation in the examined poultry farms.

*: Statistically significant at P-value < 0.05

**Table S 3.** *Escherichia coli* and *Salmonella* spp. antibiotic resistance patterns in poultry farms.

| **Antibiotic class** | **Antibiotic (µg/mL)** | **Antibiotics resistance patterns** | | | | | |
| --- | --- | --- | --- | --- | --- | --- | --- |
|  |  | ***E. coli* (%)**  **(n= 39)** | | | ***Salmonella* spp. (%)**  **(n= 23)** | | |
|  |  | **R** | **I** | **S** | **R** | **I** | **S** |
| Aminoglycosides | CN (10 μg) | 9 (23.07) | 0 (0.0) | 30 (76.9) | 8 (34.8) | 0 (0.0) | 15 (65.2) |
|  | TOB (10 μg) | 10 (25.6) | 2 (5.1) | 27 (69.2) | 11 (47.8) | 0 (0.0) | 12 (52.2) |
|  | AK (30 μg) | 11 (28.2) | 3 (7.7) | 25 (64.1) | 12 (52.2) | 0 (0.0) | 11 (47.8) |
| Carbapenems | IPM (10 μg) | 36 (92.3) | 0 (0.0) | 3 (7.7) | 0 (0.0) | 0 (0.0) | 23 (100) |
| Cephalosporines 1st & 2^nd^ generation | CFZ (30 μg) | 34 (87.2) | 0 (0.0) | 5 (12.8) | 11 (47.8) | 3 (13.1) | 9 (39.1) |
| Cephalosporines 3 rd & 4th generation | CTX (30 μg) | 9 (23.1) | 0 (0.0) | 30 (76.9) | 0 (0.0) | 0 (0.0) | 23 (100) |
|  | CAZ (10 μg) | 9 (23.1) | 0 (0.0) | 30 (76.9) | 16 (69.6) | 0 (0.0) | 7 (30.4) |
|  | FEP (30 μg) | 9 (23.1) | 0 (0.0) | 30 (76.9) | 9 (39.1) | 4 (17.4) | 10 (43.5) |
| Cephamycins | CFT (30 μg) | 34 (87.3) | 1 (2.6) | 4 (10.3) | 11 (47.8) | 0 (0.0) | 12 (52.2) |
| Quinolones | CIP (5µg) | 26 (66.7) | 3 (7.7) | 10 (25.6) | 7 (30.4) | 0 (0.0) | 16 (69.6) |
|  | NAL (30 μg) | 0 (0.0) | 14 (35.9) | 25 (64.1) | 9 (39.1) | 4 (17.4) | 10 (43.5) |
| Folate pathway inhibitors | SXT (1.25/23.75 μg) | 12 (30.8) | 0 (0.0) | 27 (69.2) | 7 (30.4) | 0 (0.0) | 16 (69.6) |
| Monobactams | ATM (30 μg) | 32 (82.1) | 0 (0.0) | 7 (17.9) | 12 (30.4) | 0 (0.0) | 11 (69.6) |
| Pencillins | AMP (10 μg) | 39 (100) | 0 (0.0) | 0 (0.0) | 23 (100) | 0 (0.0) | 0 (0.0) |
| Pencillin -β-lactamase-inhibitor combination | SAM (30 μg) | 34 (87.3) | 0 (0.0) | 5 (12.8) | 14 (60.9) | 0 (0.0) | 9 (39.1) |
|  | PPT (30 μg) | 31 (79.5) | 2 (5.1) | 6 (15.4) | 9 (39.1) | 0 (0.0) | 14 (60.9) |
| Phenicols | CHL (30 μg) | 24 (61.5) | 0 (0.0) | 15 (38.5) | 12 (52.2) | 1 (4.3) | 10 (43.5) |
| Polymyxins | CT (25 μg) | 11 (28.2) | 0 (0.0) | 28 (71.8) | 7 (30.4) | 0 (0.0) | 16 (69.6) |
| Tetracyclines | TET (30 μg) | 39 (100) | 0 (0.0) | 0 (0.0) | 21 (91.3) | 0 (0.0) | 2 (8.7) |
| Nitrofurans | NIT (300 μg) | 0 (0.0) | 0 (0.0) | 39 (100) | 0 (0.0) | 0 (0.0) | 23 (100) |
| Macrolides | AZM (30 μg) | 11 (28.2) | 2 (5.1) | 26 (66.7) | 11 (47.8) | 4 (17.4) | 8 (34.8) |

CN; gentamicin, TOB; tobramycin, AK; amikacin, PPT; piperacillin-tazobactam, IPM; imipenem, CFZ; cefazolin, CTX; cefotaxime, CAZ; ceftazidime, FEP; cefepime, CFT; cefoxitin, CIP; ciprofloxacin, NAL; nalidixic acid, SXT; trimethoprim-sulphamethoxazole, ATM; aztreonam, AMP; ampicillin, SAM; ampicillin–clavulanic acid, CHL; chloramphenicol, CT; colistin, TET; tetracycline, NIT; nitrofurantoin, AZM; azithromycin, S; sensitive, I; intermediate sensitive, R; resistant.

**Table S 4.** Bacterial growth of XDR *E. coli* and *S. enteritidis* treated with natural melanin.

| **Incubated time (h)** |  | **Bacteria counting (log 10 CFU/mL)** | | |
| --- | --- | --- | --- | --- |
|  | ***E. coli*** | **Control *E. coli*** | ***S. enteritidis*** | **Control *S. enteritidis*** |
| 0 | 8.2±0.009 | 8.2±0.009 | 8.2±0.009 | 8.2±0.009 |
| 24 | 7.9±0.03 | 9.4±0.02 | 7.9±0.05 | 10.1±0.1 |
| 48 | 6.9±0.01 | 10.4±0.01 | 6.6 ±0.08 | 10.5±0.01 |
| 72 | 5.5±0.02 | 11.4±0.08 | 5.1±0.1 | 11.03±0.6 |
| 96 | 4.8±0.007 | 11.8±0.5 | 4.6 ± 0. 2 | 11.5±0.03 |
| 120 | 0.0 | 12.4±0.02 | 0.0 | 12.4-±0.03 |


**Table S 5**. The relative m*RNA* expression levels of *E. coli* and *S. enteritidis* associated virulence genes before and after treatment with natural melanin.

| **Incubated time (h)** | ***E. coli*** | | | | ***S. enteritidis*** | | |  |
| --- | --- | --- | --- | --- | --- | --- | --- | --- |
|  | ***stx*1** | ***stx*2** | ***eae*A** | ***hyl*A** | ***sop*B** | ***stn*** | ***hil*A** | ***spv*C** |
| 24 | 0.863±0.066^a^ | 0.9±0.036^a^ | 0.613±0.02^a^ | 0.81±0.07^a^ | 0.96±0.04^a^ | 0.9±0.036^a^ | 0.446±0.037 ^a^ | 0.846±0.03 ^a^ |
| 48 | 0.663±0.066^b^ | 0.566±0.025^b^ | 0.153±0.058^a^ | 0.536±0.015^a^ | 0.73±0.055^a^ | 0.516±0.095 ^a^ | 0.153±0.058^b^ | 0.547±0.03 ^b^ |
| 72 | 0.17±0.04^c^ | 0.136±0.02^c^ | 0.0943±0.003^b^ | 0.149±0.046 ^b^ | 0.22±0.03^b^ | 0.136±0.02^b^ | 0.0443±0.012^b^ | 0.143±0.056 ^c^ |
| 96 | 0.0366±0.003^c^ | 0.034±0.004^c^ | 0.019±0.004^c^ | 0.0196±0.005^b^ | 0.077±0.002^b^ | 0.037±0.01^b^ | 0.0193± 0.004^b^ | 0.023± 0.01 ^c^ |
| 120 | 0±0.00^c^ | 0±0.00^d^ | 0±0.00^c^ | 0±0.00^b^ | 0±0.00^c^ | 0±0.00^b^ | 0±0.00^b^ | 0±0.00^c^ |
| P-value overall | 0.002* | <0.001* | 0.001* | 0.002* | 0.001* | 0.002* | 0.005* | <0.001* |

^a^ values±SD; means with different subscript letters are significantly different (*P*< 0.05), according to One-way repeated-measures ANOVA, followed by Bonferroni post-hoc comparisons.
